# Supplementary material for: Effectiveness and cost-effectiveness of the GoActive intervention to increase physical activity among UK adolescents: A cluster randomised controlled trial
Source: PLoS Med. 2020 Jul 23;17(7):e1003210. doi: 10.1371/journal.pmed.1003210 (PMC7377379; doi:10.1371/journal.pmed.1003210)
Supplement: S1 Fig — (DOCX) [file pmed.1003210.s002.docx]

## S1 Fig. GoActive tiered delivery system.

The iteratively developed, feasibility-tested and refined 12-week intervention trained older adolescents (mentors) and in-class peer leaders to encourage classes to undertake two new weekly activities. Training sessions consisted of an initial session between facilitators and mentors lasting at least 1 h followed by six 30 min meetings every week during the first six weeks and meetings approximately every 2 weeks thereafter. Mentors met with peer leaders weekly.
