# Supplementary figures and images for: Effectiveness and cost-effectiveness of the GoActive intervention to increase physical activity among UK adolescents: A cluster randomised controlled trial
Source: PLoS Med. 2020 Jul 23;17(7):e1003210. doi: 10.1371/journal.pmed.1003210 (PMC7377379; doi:10.1371/journal.pmed.1003210)

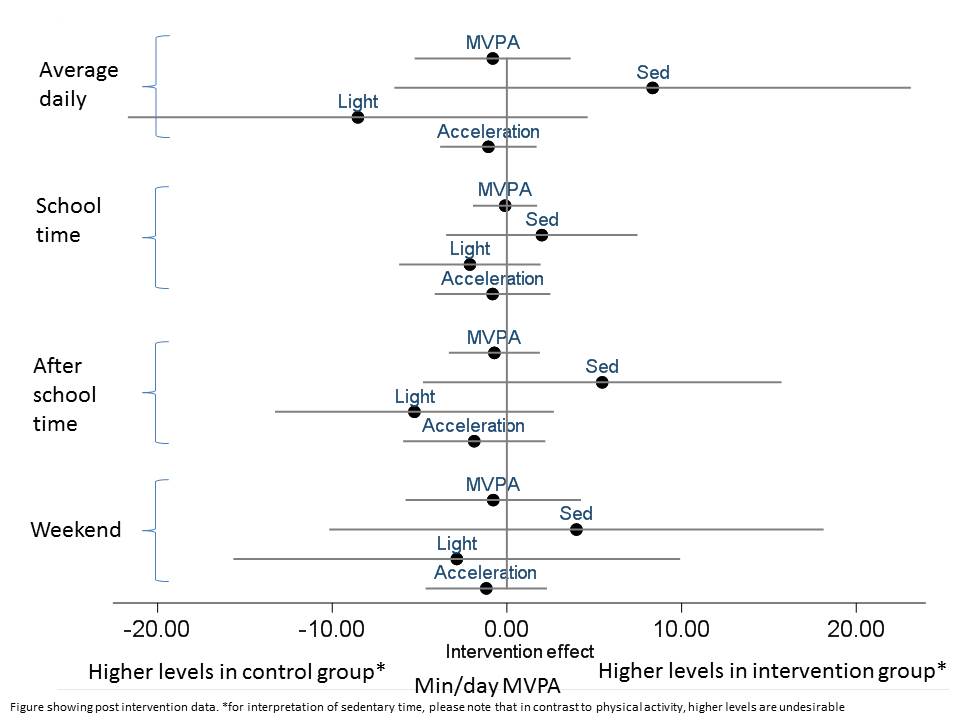


## S2 Figure. Intervention effect on continuous secondary PA outcomes at post-intervention

Supplement: S2 Fig — (DOCX) [file pmed.1003210.s003.docx]
