# Supplementary material for: Effectiveness and cost-effectiveness of the GoActive intervention to increase physical activity among UK adolescents: A cluster randomised controlled trial
Source: PLoS Med. 2020 Jul 23;17(7):e1003210. doi: 10.1371/journal.pmed.1003210 (PMC7377379; doi:10.1371/journal.pmed.1003210)
Supplement: S1 Table — (DOCX) [file pmed.1003210.s004.docx]

| S1 Table. GoActive study outcomes. | | |
| --- | --- | --- |
| **Accelerometry-derived physical activity outcomes:** Continuous waveform data from wrist-worn Axivity monitor (minutes/day) | | |
| **Primary outcome** | **Description** | **Time point** |
| *Moderate-to-vigorous physical activity (MVPA***)** | Average daily minutes | 10-months |
| **Secondary outcomes** | **Description** | **Time point** |
| *MVPA* | Average daily minutes | Post-intervention |
|  | School time (9am-3pm)  Weekdays after school (3pm onwards)  Weekends | Post-intervention , 10-months |
| *Sedentary time* | Average daily minutes  School time (9am-3pm)  Weekdays after school (3pm onwards)  Weekends | Post-intervention , 10-months |
| *Light intensity activity* | Average daily minutes  School time (9am-3pm)  Weekdays after school (3pm onwards)  Weekends | Post-intervention , 10-months |
| *Average acceleration* | Average daily acceleration in milli-g  School time (9am-3pm)  Weekdays after school (3pm onwards)  Weekends | Post-intervention , 10-months |
| **Questionnaire derived physical activity outcome at post-intervention and 10-months** | | |
| *Self-reported physical activity* | Continuous;  Self-reported participation frequency based on 28 activities from the Youth Physical Activity Questionnaire (YPAQ) [[1](#_ENREF_1)]. For each activity, participation was reported as Never, Once, 2 to 3 times, 4+ times. Participation was recoded to 0, 1, 2.5, and 4.5, respectively, and summed to obtain outcome measure. | |
| **Questionnaire derived psycho-social outcomes at post-intervention and 10-months** | | |
| *Physical activity self-efficacy* | Continuous; (range 1-6)  Mean score of 8 self-reported items from Reynolds’ Psychosocial Predictors of Physical Activity: Self-efficacy scale [[2](#_ENREF_2)] | |
| *Social support for physical activity* | Continuous; (range 1-4)  Mean score of 9 self-reported items from European Youth Heart Study [[3](#_ENREF_3)] | |
| *Friendship quality* | Continuous; (range 1-5)  Mean score of 8 self-reported items used in the ROOTS project (equally weighted) [[4](#_ENREF_4)] | |
| *Well-being* | Continuous; (range 1-5)  Mean score of self-reported items using the 14-item Edinburgh-Warwick Wellbeing Scale [[5](#_ENREF_5)] | |
| *Self esteem* | Continuous; (range 1-4)  Mean score of self-reported items using the 10-item Rosenberg Self-Esteem Scale [[6](#_ENREF_6)] | |
| **Anthropometry at 10-months** | | |
| *BMI SDS* | Continuous; z-score  Body mass index (BMI) standard deviation score (SDS) calculated from height and weight data (i.e. weight / height^2^ (kg/m^2^)) collected onsite by trained research staff. Later categorised according to age and sex standardised IOTF thresholds[[7](#_ENREF_7)] | |
| *Body fat percentage* | Continuous; %  Data collected onsite using bioelectrical impedance scales. | |
| *Waist circumference* | Continuous; whole number (cm)  Data collected onsite by trained research staff. | |

**References**

1. Corder K, van Sluijs EM, Wright A, Whincup P, Wareham NJ, Ekelund U: **Is it possible to assess free-living physical activity and energy expenditure in young people by self-report?** *The American Journal of Clinical Nutrition* 2009, **89**(3):862-870.

2. Saunders R, Pate R, Felton G, Dowda M, Weinrich M, Ward D, Parsons M, Baranowski T: **Development of questionnaires to measure psychosocial influences on children's physical activity**. *Prev Med* 1997, **26**(2):241-247.

3. Ommundsen Y, Page A, Po-Wen K, Cooper AR: **Cross-cultural, age and gender validation of a computerised questionnaire measuring personal, social and environmental associations with children's physical activity: The European Youth Heart Study**. *Int J Behav Nutr Phys Act* 2008, **5**:29.

4. Goodyer IM, Herbert J, Tamplin A, Secher SM, Pearson J: **Short-term outcome of major depression: II. Life events, family dysfunction, and friendship difficulties as predictors of persistent disorder**. *J Am Acad Child Adolesc Psychiatry* 1997, **36**(4):474-480.

5. Clarke A, Friede T, Putz R, Ashdown J, Martin S, Blake A, Adi Y, Parkinson J, Flynn P, Platt S *et al*: **Warwick-Edinburgh Mental Well-being Scale (WEMWBS): validated for teenage school students in England and Scotland. A mixed methods assessment**. *BMC Public Health* 2011, **11**:487.

6. Rosenberg M: **Conceiving the Self**. New York: Basic Books; 1979.

7. Cole TJ, Bellizzi MC, Flegal KM, Dietz WH: **Establishing a standard definition for child overweight and obesity worldwide: international survey**. *BMJ* 2000, **320**(7244):1240-1243.
