# Supplementary material for: Effectiveness and cost-effectiveness of the GoActive intervention to increase physical activity among UK adolescents: A cluster randomised controlled trial
Source: PLoS Med. 2020 Jul 23;17(7):e1003210. doi: 10.1371/journal.pmed.1003210 (PMC7377379; doi:10.1371/journal.pmed.1003210)
Supplement: S2 Table — (DOCX) [file pmed.1003210.s005.docx]

## S2 Table: Reported items of GoActive process evaluation (post-intervention questionnaires)

| **Completed by** | **Topic** | **Question** | **Answer categories** |
| --- | --- | --- | --- |
| *Yr 9 students* | GoActive sessions | During the last 2 weeks, in how many tutor times/registrations did you take part in GoActive activities? | Never, once, twice, three times, nearly every day, every day |
|  | Mentors | Programme satisfaction | Strongly agree, slightly agree, slightly disagree, strongly disagree |
|  | In-class leaders | Did you have Year 9 GoActive programme leaders in your class? | No, yes, I did not take part in GoActive |
| *Mentors* | Programme satisfaction | Being a GoActive mentor is fun | Strongly agree, slightly agree, slightly disagree, strongly disagree |
| *Teachers* | Programme satisfaction | Doing the GoActive programme is enjoyable for me to facilitate | Strongly disagree, slightly disagree, slightly agree, strongly agree |
