# Supplementary material for: Effectiveness and cost-effectiveness of the GoActive intervention to increase physical activity among UK adolescents: A cluster randomised controlled trial
Source: PLoS Med. 2020 Jul 23;17(7):e1003210. doi: 10.1371/journal.pmed.1003210 (PMC7377379; doi:10.1371/journal.pmed.1003210)
Supplement: S3 Table — (DOCX) [file pmed.1003210.s006.docx]

## S3 Table: Pattern of missing data in the primary outcome (accelerometer-assessed MVPA at 10-month follow-up).

|  | **MISSING** | | | **AVAILABLE** | | |
| --- | --- | --- | --- | --- | --- | --- |
|  | **N=988** | | | **N=1874** | | |
|  | **Mean** | **SD** |  |  | **Mean** | **SD** |
|  |  |  |  |  |  |  |
| **Age (yrs)** | 13.3 | 0.4 |  |  | 13.2 | 0.4 |
| **BMI SDS** | 0.5 | 1.2 |  |  | 0.3 | 1.2 |
| **Body fat (%)** | 20.8 | 10.0 |  |  | 20.8 | 9.9 |
| **Waist circumference (cm)** | 71.1 | 10.3 |  |  | 69.7 | 9.2 |
|  | **%** | **N** |  |  | **%** | **N** |
| **Gender** |  |  |  |  |  |  |
| *Male* | 55.5 | 548 |  |  | 50.4 | 944 |
| *Female* | 44.5 | 440 |  |  | 49.6 | 930 |
| **Weight categories** |  |  |  |  |  |  |
| *With underweight/normal weight* | 64.6 | 638 |  |  | 72.4 | 1357 |
| *With overweight/obesity* | 35.4 | 350 |  |  | 27.6 | 517 |
| **Ethnicity** |  |  |  |  |  |  |
| *White* | 83.1 | 821 |  |  | 85.5 | 1602 |
| *Mixed (identifying with multiple ethnicities)* | 6.7 | 66 |  |  | 6.0 | 113 |
| *Asian (including South-Asian and Chinese)* | 3.2 | 32 |  |  | 4.1 | 76 |
| *African and/or Caribbean* | 3.1 | 31 |  |  | 2.1 | 39 |
| *Other* | 2.0 | 20 |  |  | 1.5 | 28 |
| **Family socioeconomic status** |  |  |  |  |  |  |
| *Low* | 18.3 | 181 |  |  | 11.5 | 216 |
| *Medium* | 43.2 | 427 |  |  | 41.5 | 778 |
| *High* | 37.2 | 368 |  |  | 46.2 | 866 |
| **County** |  |  |  |  |  |  |
| *Cambridgeshire* | 38.0 | 375 |  |  | 56.2 | 1054 |
| *Essex* | 62.0 | 613 |  |  | 43.8 | 820 |

*Note:* Primary outcome (MVPA at 10-month follow-up) was missing in 448 (34.0%) control and 540 (35.0%) intervention group participants.
