# Supplementary material for: Effectiveness and cost-effectiveness of the GoActive intervention to increase physical activity among UK adolescents: A cluster randomised controlled trial
Source: PLoS Med. 2020 Jul 23;17(7):e1003210. doi: 10.1371/journal.pmed.1003210 (PMC7377379; doi:10.1371/journal.pmed.1003210)
Supplement: S4 Table — (DOCX) [file pmed.1003210.s007.docx]

## S4 Table. GoActive blinding summary

Staff involved measurements at follow-up (n=15) were asked: What schools are control? What schools are intervention? If they listed any, they were asked to state how they knew this. Please note school names are pseudonyms.

|  | Role | Confirmed school | How they found out | Staff member response to question |
| --- | --- | --- | --- | --- |
| 1 | Dedicated GoActive research assistant |  |  | If assisting with questionnaire checking (this was rare, i.e. checking <5 questionnaires at each school) I would often be asked by pupils to help explain what was meant by the question “Was my school intervention or control?”. After hearing another member of staff respond to this question by saying that “intervention schools would have had access to a website”, I adopted this habit. Participants would often reply to this by saying they did or did not remember using a website, but I never retained this information. |
| 2 | Dedicated GoActive research assistant | A (control) | I can’t remember how I found out, I think through pupils discussing. | I think I kept pretty much blinded throughout the measurement sessions. There may have been a couple of occasions when a pupil said that the school didn’t engage with any of the activities or carry on GoActive but I can’t remember which schools those pupils was from as I just tried to block anything like that out. |
|  |  | J (intervention) | The school teacher passed us lots of unused equipment (hoodies, t-shirts and the activity cards). |  |
| 3 | Dedicated GoActive research assistant | B (control) | I think the teacher or a few pupils mentioned something about not having the intervention. | There are a few schools that I think are control and intervention but only thought this after the measurement sessions had taken place. |
|  |  | J (intervention) | The teacher handed back intervention materials to us at the final session. |  |
|  |  | K (intervention) | There were boxes around the office labelled with pupil rewards given out at previous intervention stages. |  |
|  |  | L (intervention) | Pupils and the teacher were discussing the control / intervention question and spoke about the activities they had done in form time. |  |
| 4 | Unit staff employed to work on other projects (occasional support) |  |  | I cannot recall anything from my time in the schools that would indicate which arm of the study they were in. |
| 5 | Unit staff employed to work on other projects (occasional support) |  |  | I cannot tell you which Go Active schools were control or intervention schools. |
| 6 | Unit staff employed to work on other projects (occasional support) |  |  | I have no idea on either control or intervention schools. At the time of session when checking questionnaires you can see what the majority of students put for the question asking them which group they thought they were in so you can kind of assume from that. I can’t remember now what they were. |
| 7 | University Bank Staff (occasional support) |  |  | There were some schools I knew that were control because of the students lack of knowledge about the whole process at which they were undergoing/the type of questions they would ask. Intervention schools were not always as clear cut and most of the time I couldn't be entirely sure however I would base it off their knowledge of the process and the questions they would ask.    On the other hand there were schools where I had no clue, which was due to the mixed responses of the students. |
| 8 | Unit staff employed to work on other projects (occasional support) |  |  | You must have done a good job because I had no idea if the schools I attended were control or intervention. |
| 9 | Unit staff employed to work on other projects (occasional support) |  |  | I legit couldn’t even tell you the name of the schools I attended for data collection. I never asked about a school’s allocation and wasn’t told by any of the others. |
| 10 | Unit staff employed to work on other projects (occasional support) |  |  | I think I may have only come to one school during [follow-up]. I cannot even remember what school I went to, let alone if anything was mentioned about if they were control or not. I think this helped because we are at a measuring station we didn’t really get much interaction with the students to be asked any questions. So to answer you email below, I went to one school and have no idea as to whether or not this is a control or intervention school. |
| 11 | Unit staff employed to work on other projects (occasional support) |  |  | I haven’t a clue which arm any of the schools were recruited into |
| 12 | University Bank Staff (occasional support) |  |  | As far as I remember was I not aware about what schools were interventions and controls. |
| 13 | University Bank Staff (occasional support) |  |  | I don’t know which schools were controls and which intervention. |
| 14 | University Bank Staff (occasional support) | J (intervention) | The teachers gave back a lot of items (such as sweatshirts and bags) that I assume were provided to incentivize participation in the study. |  |
| 15 | University Bank Staff (occasional support) |  |  | I am unsure as to which schools were intervention or control within the study. |
