# Supplementary material for: Effectiveness and cost-effectiveness of the GoActive intervention to increase physical activity among UK adolescents: A cluster randomised controlled trial
Source: PLoS Med. 2020 Jul 23;17(7):e1003210. doi: 10.1371/journal.pmed.1003210 (PMC7377379; doi:10.1371/journal.pmed.1003210)
Supplement: S5 Table — (DOCX) [file pmed.1003210.s008.docx]

## S5 Table. GoActive trial primary and secondary outcomes at baseline, post-intervention and 10-month follow-up

|  | **Control** | | | | | | | | | **Intervention** | | | | | | | | |
| --- | --- | --- | --- | --- | --- | --- | --- | --- | --- | --- | --- | --- | --- | --- | --- | --- | --- | --- |
|  | **Baseline** | | | **Post-intervention** | | | **10-month follow-up** | | | **Baseline** | | | **Post-intervention** | | | **10-month follow-up** | | |
| **Intention to treat population** | **N** | **Mean** | **SD** | **N** | **Mean** | **SD** | **N** | **Mean** | **SD** | **N** | **Mean** | **SD** | **N** | **Mean** | **SD** | **N** | **Mean** | **SD** |
| **Average daily mins MVPA** |  |  |  |  |  |  |  |  |  |  |  |  |  |  |  |  |  |  |
| Overall | 1224 | 35.6 | 18.9 | 904 | 35.5 | 21.4 | 871 | 27.6 | 20.6 | 1414 | 35.6 | 18.3 | 1004 | 33.6 | 22.1 | 1003 | 25.6 | 21.5 |
| School time | 1176 | 13.2 | 7.1 | 770 | 13.5 | 7.3 | 657 | 11.3 | 6.9 | 1367 | 13.3 | 7.0 | 852 | 13.1 | 8.2 | 703 | 10.2 | 7.4 |
| Weekdays after school | 1176 | 18.7 | 12.5 | 770 | 21.3 | 14.2 | 655 | 17.9 | 14.0 | 1367 | 18.6 | 12.4 | 851 | 20.0 | 15.5 | 700 | 15.4 | 13.6 |
| Weekends | 1121 | 28.2 | 24.0 | 711 | 33.2 | 30.5 | 562 | 22.3 | 24.2 | 1222 | 28.4 | 24.5 | 770 | 31.2 | 28.9 | 599 | 24.0 | 29.3 |
| **Sedentary activity** |  |  |  |  |  |  |  |  |  |  |  |  |  |  |  |  |  |  |
| Overall | 1224 | 1103.8 | 61.8 | 904 | 1106.7 | 73.5 | 871 | 1147.0 | 75.5 | 1414 | 1100.6 | 60.8 | 1004 | 1115.4 | 78.8 | 1003 | 1151.7 | 85.9 |
| School time | 1176 | 243.5 | 22.1 | 770 | 243.0 | 24.0 | 657 | 254.8 | 24.9 | 1367 | 242.1 | 22.7 | 852 | 244.6 | 26.0 | 703 | 259.1 | 28.1 |
| Weekdays after school | 1176 | 331.6 | 38.8 | 770 | 321.0 | 44.1 | 655 | 335.2 | 45.6 | 1367 | 330.2 | 39.5 | 851 | 326.8 | 46.2 | 700 | 345.1 | 46.4 |
| Weekends | 1121 | 805.0 | 101.3 | 711 | 785.8 | 121.9 | 562 | 832.4 | 116.0 | 1222 | 799.7 | 100.8 | 770 | 792.6 | 121.7 | 599 | 824.7 | 138.7 |
| **Light intensity activity** |  |  |  |  |  |  |  |  |  |  |  |  |  |  |  |  |  |  |
| Overall | 1224 | 284.2 | 59.7 | 904 | 269.4 | 78.6 | 871 | 224.7 | 83.6 | 1414 | 286.6 | 57.1 | 1004 | 261.1 | 83.8 | 1003 | 214.8 | 94.1 |
| School time | 1176 | 102.5 | 18.0 | 770 | 102.2 | 20.2 | 657 | 92.2 | 21.5 | 1367 | 103.6 | 18.3 | 852 | 100.6 | 21.9 | 703 | 88.6 | 25.0 |
| Weekdays after school | 1176 | 128.1 | 31.7 | 770 | 135.0 | 37.0 | 655 | 122.4 | 38.7 | 1367 | 129.2 | 32.6 | 851 | 129.6 | 38.5 | 700 | 114.1 | 40.7 |
| Weekends | 1121 | 240.2 | 93.6 | 711 | 250.6 | 113.3 | 562 | 207.4 | 114.6 | 1222 | 244.7 | 92.3 | 770 | 245.5 | 112.1 | 599 | 212.6 | 134.8 |
| **Overall activity (average acceleration)** |  |  |  |  |  |  |  |  |  |  |  |  |  |  |  |  |  |  |
| Overall | 1176 | 42.7 | 13.2 | 770 | 43.6 | 14.1 | 657 | 37.6 | 12.7 | 1367 | 43.0 | 12.7 | 852 | 42.2 | 13.9 | 703 | 35.9 | 14.1 |
| School time | 1176 | 45.2 | 13.9 | 770 | 45.0 | 13.9 | 657 | 39.5 | 13.2 | 1367 | 45.3 | 13.5 | 852 | 43.7 | 14.8 | 703 | 36.9 | 14.0 |
| Weekdays after school | 1176 | 45.3 | 18.9 | 770 | 48.8 | 21.1 | 655 | 42.6 | 19.7 | 1367 | 44.7 | 17.7 | 851 | 45.9 | 20.8 | 700 | 38.4 | 19.2 |
| Weekends | 1121 | 34.4 | 17.8 | 711 | 37.5 | 22.1 | 562 | 28.6 | 18.5 | 1222 | 34.8 | 18.3 | 770 | 35.6 | 20.4 | 599 | 29.8 | 22.4 |
| **Self-reported physical activity** | 1311 | 16.8 | 15.0 | 1160 | 13.2 | 10.6 | 1020 | 11.6 | 10.6 | 1529 | 17.4 | 15.6 | 1267 | 14.2 | 11.3 | 1202 | 11.6 | 10.3 |
| **Psycho-social** |  |  |  |  |  |  |  |  |  |  |  |  |  |  |  |  |  |  |
| Physical activity self-efficacy | 1302 | 2.7 | 1.1 | 1160 | 2.7 | 1.1 | 1012 | 2.9 | 1.2 | 1523 | 2.7 | 1.1 | 1267 | 2.8 | 1.2 | 1201 | 2.9 | 1.2 |
| Social support for physical activity | 1304 | 2.1 | 0.6 | 1158 | 2.0 | 0.6 | 1009 | 1.8 | 0.6 | 1522 | 2.1 | 0.7 | 1264 | 2.0 | 0.7 | 1195 | 1.9 | 0.6 |
| Friendship quality | 1305 | 2.0 | 0.6 | 1158 | 2.0 | 0.7 | 1009 | 2.1 | 0.7 | 1520 | 1.9 | 0.6 | 1265 | 2.0 | 0.6 | 1196 | 2.0 | 0.6 |
| Well-being | 1303 | 3.5 | 0.7 | 1159 | 3.4 | 0.8 | 1010 | 3.3 | 0.8 | 1520 | 3.5 | 0.7 | 1265 | 3.4 | 0.8 | 1192 | 3.3 | 0.8 |
| Self esteem | 1304 | 2.0 | 0.5 | 1158 | 2.1 | 0.6 | 1010 | 2.2 | 0.6 | 1522 | 2.0 | 0.5 | 1265 | 2.1 | 0.6 | 1192 | 2.1 | 0.6 |
| **Anthropometry** |  |  |  |  |  |  |  |  |  |  |  |  |  |  |  |  |  |  |
| BMI SDS | 1319 | 0.2 | 1.6 |  |  |  | 990 | 0.2 | 1.7 | 1543 | 0.1 | 1.9 |  |  |  | 1147 | 0.1 | 1.9 |
| Body fat (%) | 1267 | 20.7 | 10.0 |  |  |  | 903 | 20.7 | 10.4 | 1460 | 20.9 | 9.9 |  |  |  | 1022 | 20.6 | 10.3 |
| Waist circumference (cm) | 1313 | 70.0 | 9.6 |  |  |  | 974 | 72.2 | 9.3 | 1534 | 70.4 | 9.7 |  |  |  | 1119 | 72.5 | 8.9 |

Physical activity variables are accelerometry-derived outcomes; School time is 9am-3pm; Weekdays after school is from 3pm.
