# Supplementary material for: Effectiveness and cost-effectiveness of the GoActive intervention to increase physical activity among UK adolescents: A cluster randomised controlled trial
Source: PLoS Med. 2020 Jul 23;17(7):e1003210. doi: 10.1371/journal.pmed.1003210 (PMC7377379; doi:10.1371/journal.pmed.1003210)
Supplement: S6 Table — (DOCX) [file pmed.1003210.s009.docx]

## S6 Table. Secondary outcome results for the GoActive trial average daily physical activity (minutes/day) at post-intervention.

|  | **Post intervention** | | | | | | | |
| --- | --- | --- | --- | --- | --- | --- | --- | --- |
|  | **CONTROL** | | **INTERVENTION** | | **INTERVENTION vs CONTROL** | | | |
|  | **Mean** | **SD** | **Mean** | **SD** | **Difference** | **95% CI** | | **Model N** |
|  |  |  |  |  |  |  |  |  |
| **Accelerometer assessed physical activity** |  |  |  |  |  |  |  |  |
| Average daily minutes of MVPA |  |  |  |  |  |  |  |  |
| *Overall* | -0.81 | 18.89 | -1.70 | 19.88 | -0.81 | -5.28 | 3.66 | 1908 |
| *During school time* | 0.22 | 6.16 | 0.16 | 7.22 | -0.11 | -1.94 | 1.73 | 1622 |
| *During weekdays after school* | 2.31 | 13.19 | 1.87 | 14.91 | -0.72 | -3.33 | 1.89 | 1621 |
| *At weekends* | 4.17 | 30.88 | 3.31 | 29.28 | -0.79 | -5.81 | 4.24 | 1481 |
| Average daily minutes of sedentary time |  |  |  |  |  |  |  |  |
| *Overall* | 6.34 | 70.27 | 16.17 | 74.01 | 8.34 | -6.44 | 23.13 | 1908 |
| *During school time* | -0.45 | 22.68 | 2.58 | 24.98 | 2.00 | -3.49 | 7.48 | 1622 |
| *During weekdays after school* | -8.76 | 42.04 | -2.74 | 44.02 | 5.46 | -4.81 | 15.72 | 1621 |
| *At weekends* | -14.51 | 134.20 | -3.43 | 128.46 | 3.98 | -10.17 | 18.13 | 1481 |
| Average daily minutes of light intensity physical activity |  |  |  |  |  |  |  |  |
| *Overall* | -19.84 | 71.82 | -30.05 | 76.11 | -8.54 | -21.70 | 4.63 | 1908 |
| *During school time* | -0.41 | 19.12 | -3.63 | 21.17 | -2.12 | -6.17 | 1.93 | 1622 |
| *During weekdays after school* | 4.97 | 34.38 | -1.21 | 36.32 | -5.30 | -13.28 | 2.69 | 1621 |
| *At weekends* | 3.40 | 123.82 | -7.49 | 118.43 | -2.87 | -15.66 | 9.91 | 1481 |
| Overall activity (average acceleration in milli-g) |  |  |  |  |  |  |  |  |
| *Overall* | 0.81 | 12.08 | -0.35 | 12.45 | -1.06 | -3.83 | 1.70 | 1622 |
| *During school time* | -0.36 | 12.52 | -1.08 | 13.82 | -0.82 | -4.13 | 2.49 | 1622 |
| *During weekdays after school* | 2.79 | 19.96 | 1.55 | 20.36 | -1.87 | -5.95 | 2.20 | 1621 |
| *At weekends* | 2.19 | 23.26 | 0.52 | 21.50 | -1.18 | -4.66 | 2.29 | 1481 |

Physical activity variables are accelerometry-derived outcomes; School time is 9am-3pm; Weekdays after school is from 3pm.
