# Supplementary material for: Effectiveness and cost-effectiveness of the GoActive intervention to increase physical activity among UK adolescents: A cluster randomised controlled trial
Source: PLoS Med. 2020 Jul 23;17(7):e1003210. doi: 10.1371/journal.pmed.1003210 (PMC7377379; doi:10.1371/journal.pmed.1003210)
Supplement: S7 Table — (DOCX) [file pmed.1003210.s010.docx]

## S7 Table. Secondary outcome results for the GoActive trial average daily physical activity (minutes/day) at 10-month follow-up.

|  | **10-month follow-up** | | | | | | | |
| --- | --- | --- | --- | --- | --- | --- | --- | --- |
|  | **CONTROL** | | **INTERVENTION** | | **INTERVENTION vs CONTROL** | | | |
|  | **Mean** | **SD** | **Mean** | **SD** | **Difference** | **95% CI** | | **Model N** |
|  |  |  |  |  |  |  |  |  |
| **Accelerometer assessed physical activity** |  |  |  |  |  |  |  |  |
| Average daily minutes of MVPA |  |  |  |  |  |  |  |  |
| *Overall* | -8.26 | 19.33 | -10.44 | 22.75 | -1.91 | -5.53 | 1.70 | 1874 |
| *During school time* | -1.59 | 6.30 | -2.74 | 8.02 | -1.17 | -2.74 | 0.41 | 1360 |
| *During weekdays after school* | -0.37 | 14.04 | -2.71 | 16.10 | -2.43 | -4.96 | 0.11 | 1355 |
| *At weekends* | -5.25 | 26.44 | -2.73 | 32.19 | 2.96 | -1.05 | 6.96 | 1161 |
| Average daily minutes of sedentary time |  |  |  |  |  |  |  |  |
| *Overall* | 44.31 | 78.11 | 52.78 | 88.19 | 5.34 | -7.59 | 18.27 | 1874 |
| *During school time* | 10.30 | 23.60 | 16.14 | 29.55 | 5.14 | 0.82 | 9.46 | 1360 |
| *During weekdays after school* | 3.60 | 46.62 | 12.90 | 51.13 | 9.52 | 0.52 | 18.51 | 1355 |
| *At weekends* | 30.97 | 126.85 | 23.57 | 148.55 | -13.43 | -31.81 | 4.95 | 1161 |
| Average daily minutes of light intensity physical activity |  |  |  |  |  |  |  |  |
| *Overall* | -62.87 | 82.82 | -73.35 | 93.02 | -9.46 | -23.35 | 4.44 | 1874 |
| *During school time* | -9.81 | 20.48 | -14.52 | 25.45 | -4.15 | -7.17 | -1.13 | 1360 |
| *During weekdays after school* | -6.76 | 39.22 | -13.94 | 42.03 | -7.62 | -13.72 | -1.52 | 1355 |
| *At weekends* | -40.12 | 121.98 | -34.71 | 139.19 | 11.86 | -3.39 | 27.11 | 1161 |
| Overall activity (average acceleration in milli-g) |  |  |  |  |  |  |  |  |
| *Overall* | -4.29 | 11.92 | -6.37 | 15.33 | -1.94 | -4.94 | 1.06 | 1360 |
| *During school time* | -4.94 | 12.59 | -7.47 | 15.41 | -2.60 | -5.47 | 0.26 | 1360 |
| *During weekdays after school* | -1.93 | 20.18 | -5.55 | 22.73 | -3.99 | -7.83 | -0.15 | 1355 |
| *At weekends* | -5.73 | 20.52 | -4.14 | 24.63 | 2.29 | -0.66 | 5.24 | 1161 |

Physical activity variables are accelerometry-derived outcomes; School time is 9am-3pm; Weekdays after school is from 3pm.
