# Supplementary material for: Effectiveness and cost-effectiveness of the GoActive intervention to increase physical activity among UK adolescents: A cluster randomised controlled trial
Source: PLoS Med. 2020 Jul 23;17(7):e1003210. doi: 10.1371/journal.pmed.1003210 (PMC7377379; doi:10.1371/journal.pmed.1003210)
Supplement: S8 Table — (DOCX) [file pmed.1003210.s011.docx]

## S8 Table. Secondary outcome results for the GoActive trial on psychosocial and anthropometric outcomes

|  | **CONTROL** | | **INTERVENTION** | | **INTERVENTION vs CONTROL** | | | |
| --- | --- | --- | --- | --- | --- | --- | --- | --- |
|  | **Mean** | **SD** | **Mean** | **SD** | **Difference** | **95% CI** | | **Model N** |
| **Post-intervention** |  |  |  |  |  |  |  |  |
| Self-reported physical activity | -3.46 | 13.88 | -3.43 | 13.47 | 0.62 | -0.15 | 1.38 | 2427 |
| **Psychosocial** |  |  |  |  |  |  |  |  |
| Physical activity self-efficacy | 0.04 | 0.95 | 0.16 | 0.97 | 0.12 | 0.02 | 0.23 | 2427 |
| Social support for physical activity | -0.12 | 0.55 | -0.13 | 0.56 | 0.00 | -0.06 | 0.06 | 2422 |
| Friendship quality | 0.07 | 0.62 | 0.09 | 0.57 | 0.00 | -0.05 | 0.05 | 2423 |
| Well-being | -0.02 | 0.72 | -0.09 | 0.74 | -0.04 | -0.11 | 0.02 | 2424 |
| Self esteem | 0.01 | 0.46 | 0.03 | 0.48 | 0.02 | -0.02 | 0.06 | 2423 |
|  |  |  |  |  |  |  |  |  |
| **10-month follow-up** |  |  |  |  |  |  |  |  |
| Self-reported physical activity | -5.10 | 15.60 | -5.42 | 15.37 | 0.06 | -1.03 | 1.15 | 2222 |
| **Psychosocial** |  |  |  |  |  |  |  |  |
| Physical activity self-efficacy | 0.17 | 1.12 | 0.24 | 1.06 | 0.05 | -0.03 | 0.14 | 2213 |
| Social support for physical activity | -0.22 | 0.59 | -0.22 | 0.60 | 0.02 | -0.04 | 0.09 | 2204 |
| Friendship quality | 0.11 | 0.71 | 0.09 | 0.61 | -0.04 | -0.14 | 0.06 | 2205 |
| Well-being | -0.20 | 0.83 | -0.19 | 0.78 | 0.03 | -0.07 | 0.13 | 2202 |
| Self esteem | 0.11 | 0.55 | 0.08 | 0.51 | -0.03 | -0.10 | 0.05 | 2202 |
| **Anthropometry** |  |  |  |  |  |  |  |  |
| BMI SDS | 0.05 | 0.49 | 0.05 | 0.51 | 0.01 | -0.05 | 0.07 | 2089 |
| Body fat (%) | 0.01 | 4.56 | 0.30 | 4.48 | 0.26 | -0.94 | 1.46 | 1925 |
| Waist circumference (cm) | 2.41 | 4.91 | 2.86 | 4.57 | 0.48 | -0.19 | 1.15 | 2093 |

Intervention effect is the difference in mean change (baseline to post-intervention, or baseline to 10-month follow-up) in outcome (adjusted for baseline) between the intervention and control group. Difference is estimated from a linear regression model, including parameters for randomised group (control, intervention), baseline value of the outcome (i.e. Analysis of Covariance), pupil premium (low, high), and county (Cambridgeshire, Essex). Robust standard errors were calculated to allow for non-independence of individuals within schools. Missing indicator method is used to enable participants with a missing baseline value of the outcome to be included in the analysis. Participants with a missing value of the outcome at the relevant follow-up visit (either post-intervention or 10-month follow-up) are excluded from this analysis. Anthropometry was not assessed post-intervention
