# Supplementary material for: Effectiveness and cost-effectiveness of the GoActive intervention to increase physical activity among UK adolescents: A cluster randomised controlled trial
Source: PLoS Med. 2020 Jul 23;17(7):e1003210. doi: 10.1371/journal.pmed.1003210 (PMC7377379; doi:10.1371/journal.pmed.1003210)
Supplement: S9 Table — (DOCX) [file pmed.1003210.s012.docx]

## S9 Table. Effect modification of the primary outcome, average minutes of MVPA/day.

|  |  | **95% CI** | |  |  |  |  |
| --- | --- | --- | --- | --- | --- | --- | --- |
| Interaction between randomised group and.... | **estimate** | **lower** | **upper** | **p-value** |  |  |  |
|  |  |  |  |  |  |  |  |
| Sex (boys vs girls) | -2.97 | -5.24 | -0.70 | 0.022 |  |  |  |
| Socioeconomic status (medium/low vs high) | -6.89 | -11.04 | -2.74 | 0.005 |  |  |  |
| Ethnicity (White vs any other ethnicity) | -1.81 | -5.33 | 1.71 | 0.330 |  |  |  |
| Baseline physical activity (≥60 mins average daily MVPA vs <60 mins) | -6.04 | -17.52 | 5.43 | 0.318 |  |  |  |
| Weight status (with underweight/normal weight vs with overweight/obesity) | 4.64 | -0.15 | 9.43 | 0.077 |  |  |  |
|  |  |  |  |  |  |  |  |
|  |  |  |  |  |  |  |  |
| **Subgroup analyses** |  |  |  |  |  |  |  |
|  | **10-month follow-up – baseline** | | | | | | |
|  | **CONTROL** | | **INTERVENTION** | | **INTERVENTION vs CONTROL** | | |
|  | **Mean** | **SD** | **Mean** | **SD** | **Difference** | **95% CI** | |
|  |  |  |  |  |  |  |  |
| **Sex** |  |  |  |  |  |  |  |
| *Boys* | -10.15 | 22.30 | -13.77 | 24.94 | -3.44 | -7.42 | 0.54 |
| *Girls* | -6.43 | 15.75 | -7.20 | 19.88 | -0.20 | -3.56 | 3.16 |
| **Socioeconomic status** |  |  |  |  |  |  |  |
| *Medium/low* | -10.07 | 18.46 | -2.71 | 21.67 | 4.25 | -0.66 | 9.16 |
| *High* | -8.04 | 19.47 | -11.54 | 22.79 | -2.72 | -6.33 | 0.89 |
| **Ethnicity** |  |  |  |  |  |  |  |
| *White* | -4.62 | 19.04 | -8.07 | 24.83 | -1.08 | -5.06 | 2.89 |
| *Any other ethnicity* | -8.66 | 19.39 | -10.91 | 22.46 | -2.18 | -5.97 | 1.62 |
| **Baseline physical activity** |  |  |  |  |  |  |  |
| *≥60 mins average daily MVPA* | -5.92 | 16.25 | -7.83 | 20.11 | -1.45 | -4.77 | 1.87 |
| *<60 mins average daily MVPA* | -29.10 | 29.59 | -36.08 | 30.19 | -6.51 | -16.40 | 3.37 |
| **Weight status** |  |  |  |  |  |  |  |
| *With underweight/normal weight* | -8.58 | 20.30 | -11.58 | 22.30 | -3.14 | -7.02 | 0.74 |
| *With overweight/obesity* | -7.39 | 16.43 | -7.42 | 23.66 | 0.99 | -3.80 | 5.79 |
|  |  |  |  |  |  |  |  |
| Interactions between randomised group and each potential moderator are estimated by including the relevant interaction parameter(s) in the ANCOVA model used in the primary outcome analysis. | | | | | | | |
| For each interaction, the p-value is from an F-test of the null hypothesis that the true interaction parameter(s)=0. | | | | |  |  |  |
