# Supplementary material for: Effectiveness and cost-effectiveness of the GoActive intervention to increase physical activity among UK adolescents: A cluster randomised controlled trial
Source: PLoS Med. 2020 Jul 23;17(7):e1003210. doi: 10.1371/journal.pmed.1003210 (PMC7377379; doi:10.1371/journal.pmed.1003210)
Supplement: S10 Table — (DOCX) [file pmed.1003210.s013.docx]

## S10 Table. Primary outcome of the GoActive trial, average minutes of MVPA/day by per protocol population.

| **Control** | | | **Intervention** | | | **Intervention vs Control** |
| --- | --- | --- | --- | --- | --- | --- |
|  | | |  | | |  |
| **Baseline** | **10-month follow-up** | **Change from baseline** | **Baseline** | **10-month follow-up** | **Change from baseline** | **Difference (95% CI)** |
| N=1224 | N=871 |  | N=365 | N=285 |  |  |
|  |  |  |  |  |  |  |
| 35.6 (18.9) | 27.6 (20.6) | -8.3 (19.3) | 36.9 (19.1) | 25.9 (20.7) | -11.1 (23.3) | -1.87 (-6.80, 3.06) |

Values are Mean (SD). Per protocol population defined as reporting being active during tutor times at least twice during the last two weeks (as assessed in week 6 of the intensely facilitated phase of the intervention) *and* logging activity points on the website at least once during the whole intervention period.
