# Supplementary material for: Effectiveness and cost-effectiveness of the GoActive intervention to increase physical activity among UK adolescents: A cluster randomised controlled trial
Source: PLoS Med. 2020 Jul 23;17(7):e1003210. doi: 10.1371/journal.pmed.1003210 (PMC7377379; doi:10.1371/journal.pmed.1003210)
Supplement: S11 Table — (DOCX) [file pmed.1003210.s014.docx]

## S11 Table. Post-hoc sensitivity analyses with different pre-processing decisions regarding primary outcome data

|  | **Control** | | | **Intervention** | | | **Intervention vs Control** |
| --- | --- | --- | --- | --- | --- | --- | --- |
|  |  | | |  | | |  |
|  | **Baseline** | **10-month follow-up** | **Change from baseline** | **Baseline** | **10-month follow-up** | **Change from baseline** | **Difference (95% CI)** |
|  |  |  |  |  |  |  |  |
| **Night time excluded from pre-processing** | N=1176 | N=657 |  | N=1367 | N=703 |  | N=1360 |
|  | 37.1 (19.9) | 31.9 (19.3) | -4.1 (17.3) | 37.3 (19.5) | 29.7 (22.0) | -6.7 (23.4) | -2.52 (-7.33, 2.29) |
|  |  |  |  |  |  |  |  |
| **Using 12 hours of wear per quadrant** | N=1097 | N=537 |  | N=1262 | N=535 |  | N=1072 |
|  | 36.8 (19.2) | 31.8 (18.2) | -3.4 (15.4) | 36.9 (18.9) | 31.5 (22.2) | -3.4 (21.4) | -0.15 (-5.25, 4.95) |

Intervention effect is the difference in mean change (Baseline to 10-month follow-up) in average daily minutes of MVPA (adjusted for baseline) between the intervention and control group. Difference is estimated from a linear regression model, including parameters for randomised group (control, intervention), baseline value of the outcome (i.e. Analysis of Covariance), pupil premium (low, high), and county (Cambridgeshire, Essex). Robust standard errors were calculated to allow for non-independence of individuals within schools. Missing indicator method is used to enable participants with a missing baseline value of the outcome to be included in the analysis. Participants with a missing value of the outcome at follow-up (10-month follow-up) are excluded from this analysis.
