# Supplementary material for: Effectiveness and cost-effectiveness of the GoActive intervention to increase physical activity among UK adolescents: A cluster randomised controlled trial
Source: PLoS Med. 2020 Jul 23;17(7):e1003210. doi: 10.1371/journal.pmed.1003210 (PMC7377379; doi:10.1371/journal.pmed.1003210)
Supplement: S12 Table — (DOCX) [file pmed.1003210.s015.docx]

## S12 Table. Protocol-based costing per school per year.

|  | **Unit cost** | **units** | **Quantity** | **Total** | **Unit cost source / notes** |
| --- | --- | --- | --- | --- | --- |
| **Facilitator time** |  |  |  |  |  |
| facilitator training day | £15.26 | hour | 8 | £122.05 | Hourly rate assuming 46 working weeks of 37.5hours.^a^ |
| 2 hrs per week per school x 12 weeks | £15.26 | hour | 24 | £366.15 |  |
| facilitator training school staff | £15.26 | hour | 8 | £122.05 |  |
|  |  |  |  |  |  |
| **Materials** |  |  |  |  |  |
| Activity cards | £5.00 | - | 1 | £5.00 | representative cost |
| Facilitator training manual | £5.00 | - | 1 | £5.00 | representative cost |
|  |  |  |  |  |  |
| **Teacher time** |  |  |  |  |  |
| training day (led by facilitator) | £17.26 | hour | 32 | £552.44 | Assuming 4 teachers being trained, 8 hour day.^b^ |
| 30 mins per week | £17.26 | hour | 78 | £1,346.57 | Assuming 46 working weeks of 37.5 hours, 39 weeks in school year = 19.5 hours of teacher time x 4 teachers.^c^ |
|  |  |  |  | £2519.26 |  |

^a^ <https://neu.org.uk/advice/support-staff-pay-and-conditions>

^b^ <https://www.nasuwt.org.uk/advice/pay-pensions/pay-scales/england-pay-scales.html>

^c^ <https://www.nasuwt.org.uk/advice/pay-pensions/pay-scales/england-pay-scales.html>
