# Supplementary material for: Effectiveness and cost-effectiveness of the GoActive intervention to increase physical activity among UK adolescents: A cluster randomised controlled trial
Source: PLoS Med. 2020 Jul 23;17(7):e1003210. doi: 10.1371/journal.pmed.1003210 (PMC7377379; doi:10.1371/journal.pmed.1003210)
Supplement: S13 Table — (DOCX) [file pmed.1003210.s016.docx]

## S13 Table. Conversion from cost per school to cost per student

| Cost per school: | £2,519.26 |
| --- | --- |
| N schools: | 8 |
| N students: | 1543 |
| £ per student: | £13.06 |
