# Supplementary material for: Effectiveness and cost-effectiveness of the GoActive intervention to increase physical activity among UK adolescents: A cluster randomised controlled trial
Source: PLoS Med. 2020 Jul 23;17(7):e1003210. doi: 10.1371/journal.pmed.1003210 (PMC7377379; doi:10.1371/journal.pmed.1003210)
Supplement: S14 Table — (DOCX) [file pmed.1003210.s017.docx]

## S14 Table. Quality of life (assessed with CHU-9D) based quality-adjusted life years (QALYs) gained.

| Group | n | QALYs gained (undiscounted) | QALYs gained (discounted) |
| --- | --- | --- | --- |
| 0 | 885 | 1.258 (0.005) | 1.244 (0.005) |
| 1 | 919 | 1.256 (0.005) | 1.242 (0.005) |
| Increment |  | -0.004 (0.006) | -0.004 (0.006) |
| Increment* |  | -0.006 (0.006) | -0.006 (0.006) |

* Adjusted for baseline covariates with missing data imputed using multiple imputation.
