# Supplementary material for: Effectiveness and cost-effectiveness of the GoActive intervention to increase physical activity among UK adolescents: A cluster randomised controlled trial
Source: PLoS Med. 2020 Jul 23;17(7):e1003210. doi: 10.1371/journal.pmed.1003210 (PMC7377379; doi:10.1371/journal.pmed.1003210)
Supplement: S1 Text — (DOCX) [file pmed.1003210.s018.docx]

## S1 Text. Key elements of GoActive intervention

The intervention programme comprised components across six main themes:

1. Choice: Each tutor group chose two different activities weekly which they participated in as a class.
2. Novelty: 21 (mostly unusual) activities were available and designed to utilise little or no equipment.
3. Mentorship: Older adolescents in the school (mentors) paired with each Year 9 class to encourage participation in new activities. Mentors were helped by Year 9 in-class leaders (a boy and a girl) who changed weekly.
4. Competition: Students gained activity points every time they recorded doing an activity; there was no expectation of duration or intensity, students just had to try an activity to get points. Individual points were kept private with class level totals announced to encourage inter-class competition. Points were logged on the study website by students.
5. Rewards: Students gained small individual prizes for reaching certain points levels; thresholds were graded with decreasing frequency.
6. Flexibility: at least one tutor time per week was used to do an activity and participants were also encouraged to do activities at other times, especially out of school.
