# Supplementary material for: Effectiveness and cost-effectiveness of the GoActive intervention to increase physical activity among UK adolescents: A cluster randomised controlled trial
Source: PLoS Med. 2020 Jul 23;17(7):e1003210. doi: 10.1371/journal.pmed.1003210 (PMC7377379; doi:10.1371/journal.pmed.1003210)
Supplement: S2 Text — (DOCX) [file pmed.1003210.s019.docx]

## S2 Text: Impact of deviations from the missing at random assumption on the results for the primary outcome.

The primary analysis assumes that missing data in MVPA at 10-month follow-up are missing at random. The graph below shows the impact of deviations from this assumption on the estimated intervention effect, based on a pattern mixture analysis [[1](#_ENREF_1)] using the “rctmiss” command in Stata. The results show that even when those with missing data could be assumed to do at least 10 minutes more or 10 minutes less MVPA than those with available data, the intervention effects and 95% CIs are very similar to those in the primary analysis.

1. White IR, Carpenter J, Horton NJ: **Including all individuals is not enough: lessons for intention-to-treat analysis**. *Clinical trials* 2012, **9**(4):396-407.
