# Supplementary material for: Effectiveness and cost-effectiveness of the GoActive intervention to increase physical activity among UK adolescents: A cluster randomised controlled trial
Source: PLoS Med. 2020 Jul 23;17(7):e1003210. doi: 10.1371/journal.pmed.1003210 (PMC7377379; doi:10.1371/journal.pmed.1003210)
Supplement: Attachment — Submitted filename: corder.pdf [file pmed.1003210.s020.pdf]

This paper reports a cluster trial of an intervention to promote physical activity in young people.

I was asked for a statistical report and I interpret that to include all aspects of the design and conduct of the study. The version I received did not have page numbers. My numbering has the abstract starting on page 2

Points of detail

Page 8 What is BMI SDS? I know what BMI is obviously. Is this waht the text refers to as a z–score?

Page 9 If I understand the imputation correctly this was a form of single imputation. Since substantial proportions seem to be missing at 10 months this seems unwise as it will underestimate the variability. Some form of multiple imputation would be much better and also some expansion of the description here especially if I have not understood correctly.

Page 10 Categorising an essentially continuous variable like socio–economic status wastes information (Altman and Royston, 2006; Royston et al., 2006) and leads to models which are often implausible as they predict the effect remaining flat within categories and then jumping to a new value at the category boundary. Surely a continuous variable could have been used in the interaction term?

Page 10 I did not see the secondary outcomes described in Table 1. In fact they only seem to appear in Figure 3.

Page 12 I would have thought the outcome here was ordered so an ordinal regression would have been more appropriate than a multinomial.

Page 16 The baseline N is larger than the 10 month N so the means are obviously based on different samples. I wonder whether this is helpful as the full baseline sample is not contributing to the change score. We do have the change score with its confidence interval of course so this may not matter too much.

Page 17, Figure 4 Medium/low v high is presumably socio–economic position but that could be made explicit.

Page 18 I did not understand the sentence starting Observation contradicted.

Page 19 I think one of the references to per protocol should be to intention to treat.

Page 23 Perhaps ‘largest to our knowledge’ would be safer.

Page 34, Figure 2 This figure could do with more explanation. Is acceleration measured in units of minutes per day as the caption suggests? It is usually length per time squared. We could also use a description of the exact time periods here as well as in the text

Page 35, Figure 3 Why do we see the post intervention results here for the secondary outcomes but not, as far as I can see, for the primary?

Points of more substance

Detail of reporting

The authors make copious reference to thei published protocol. I feel that we need more of the detail from that in this paper too. At the moment it is hard in places with awkward cross-referencing. A particular issue is the secondary outcomes which are really obscure. I think we need to know what scale was used to measure each and at least some indication of the ranges of scores to interpret the x–axis in Figure 3. I know some of this detail is in the supplement but surely the outcomes are important enough for the main paper.

The discussion makes reference in several places to the outcome of the process evaluation but this is still in preparation if I read the statement correctly. It does seem a shame to have the results of a mixed–methods study split like this although I know it is often done.

Interpretation

Since the study did show at least some effects in the moderator analyses the treatment must have had some effect even if not the desired one. Moderator effects being interaction are less precisely estimated as well which strengthens the point. Does this affect our view of what the study actually found?

Summary

Mostly about presentation and some suggestions for enhanced analysis.

Michael Dewey

References

D G Altman and P Royston. The cost of dichotomising continuous variables. *British Medical Journal*, 332:1080, 2006.

P Royston, D G Altman, and W Sauerbrei. Dichotomizing continuous predictors in multiple regression: a bad idea. *Statistics in Medicine*, 25:127–141, 2006.
