# Supplementary material for: Effectiveness and cost-effectiveness of the GoActive intervention to increase physical activity among UK adolescents: A cluster randomised controlled trial
Source: PLoS Med. 2020 Jul 23;17(7):e1003210. doi: 10.1371/journal.pmed.1003210 (PMC7377379; doi:10.1371/journal.pmed.1003210)
Supplement: Attachment — Submitted filename: Corder_PlosMed_R2_response.docx [file pmed.1003210.s021.docx]

**RESPONSE TO COMMENTS**

**Editor comments:**

**Abstract**

** Please structure your abstract using the PLOS Medicine headings (Background, Methods and Findings, Conclusions).*

Response: This has been revised.

** Please combine the Methods and Findings sections into one section, “Methods and findings”.*

Response: this has been revised

*Background- please briefly mention reasons for low physical activity in adolescents*

Response: The determinants of the decline, and low levels, of physical activity in adolescence are complex and multi-level, and generally poorly understood. However, the school social and physical environment is likely to play a role, and is therefore the target of the GoActive intervention. We have included the following (p2, line 34-36): “The determinants of physical activity in adolescents are multilevel and poorly understood, but the school’s social environment likely plays an important role.”

*Methods Please clarify 84% eligible students?*

Response: We have add “of” (84% of eligible students).

*Please provide additional demographics- like gender?*

Response: we have included % male on p2, line 55 (52.1% male)

*Please clarify if both Cambridgeshire and Essex schools participated and consider revising the sentence for clarity.*

Response: We have revised on p2, line 42-43: “Non-fee-paying, co-educational schools including Year 9 students in the UK counties of Cambridgeshire and Essex were eligible for inclusion”

*In the last sentence of the Abstract Methods and Findings section, please describe the main limitation(s) of the study's methodology.*

Response: We have added the following sentence on p3, line 62-64: “The main methodological limitation of GoActive was the relatively affluent and ethnically homogeneous sample.”

*Please replace the interpretation section with conclusions Abstract Conclusions:*

Response: this has been revised and now reads (p3, line 67-72):

“In this study, we observed that a rigorously developed school-based intervention was no more effective than standard school practice at preventing declines in adolescent physical activity. Interdisciplinary research is required to understand educational setting-specific implementation challenges. School-leaders and authorities should be realistic about expectations of effect of school-based physical activity promotion strategies implemented at scale.”

** Please address the study implications without overreaching what can be concluded from the data; the phrase "In this study, we observed ..." may be useful.*

** Please interpret the study based on the results presented in the abstract, emphasizing what is new without overstating your conclusions.*

** Please avoid vague statements such as "these results have major implications for policy/clinical care". Mention only specific implications substantiated by the results.*

** Please avoid assertions of primacy ("We report for the first time....")*

Response to all above comments: The Abstract conclusions have been revised and now reads (p3, line 67-72):

“In this study, we observed that a rigorously developed school-based intervention was no more effective than standard school practice at preventing declines in adolescent physical activity. Interdisciplinary research is required to understand educational setting-specific implementation challenges. School-leaders and authorities should be realistic about expectations of effect of school-based physical activity promotion strategies implemented at scale.”

*Funding information should be moved to the funding section of the article meta-data*

Response: Funding information has been moved to the relevant section at the end of the main manuscript.

*The Data Availability Statement (DAS) requires revision.*

Response: We have amended the DAS as requested.

*At this stage, we ask that you include a short, non-technical Author Summary of your research to make findings accessible to a wide audience that includes both scientists and non-scientists. The Author Summary should immediately follow the Abstract in your revised manuscript. This text is subject to editorial change and should be distinct from the scientific abstract. Please see our author guidelines for more information:* [*https://journals.plos.org/plosmedicine/s/revising-your-manuscript#loc-author-summary*](https://journals.plos.org/plosmedicine/s/revising-your-manuscript#loc-author-summary)

Response: We have now included an author summary (p4).

*Introduction*

References- please use square brackets throughout

Response: this has been revised throughout.

*“Compared to their inactive peers, active adolescents are more likely to become active, healthy and successful adults”- I would recommend removing the word successful here. Your previous work is related to education so please rephrase.*

Response: this has been revised to: “active adolescents are more likely to become active and healthy adults” (p5, line 104)

*Please include this information in the results section “Although the feasibility and pilot studies were not powered to determine effectiveness, preliminary effectiveness for our intervention was suggested, with between-arm differences in objectively measured physical activity at follow-up (difference in means 5.1 min/day (95% CI 1.1 to 9.2) (20).”*

Response: This information is related to previous published work on the GoActive intervention and these feasibility and pilot studies informed the work presented here, but were separate projects. We believe that this information should be presented in the background to the main trial (as presented here), and not as results. However, to avoid the confusion, we have removed the reference to the effect estimate referred to in the comment.

*This information ought to be in the methods and/or discussion sections “The trial reported here overcame the limitations of much previous research (15) including iterative development with the target group and school stakeholders, a sample size large enough to detect a 5-minute difference in the primary outcome between intervention and control groups, and assessment of long term effects of device-measured physical activity”*

Response: We agree, and this has now been moved to the Strengths and Limitations section of the discussion (p23/24).

*Methods*

*Could you please mention if the passive mode of parental l consent was approved by the ethics committee?*

Response: this has been changed and now reads: “Ethical approval was obtained from the University of Cambridge Psychology Ethics Committee (PRE.126.2016), and included approval to obtain passive parental consent and written student assent for study participation” (p6, 149-152)

*Role of the funding source- please move this information to the financial disclosure section*

Response: This has been deleted as a statement to this effect was already included in the funding section.

*Please ensure all questionnaires are cited or provided as SI files.*

Response: we have checked the text and all outcome questionnaires are cited. We have added a table to the supplementary file (S3 Table) presenting the process evaluation questions included in the current manuscript.

*Discussion*

*Please present and organize the Discussion as follows: a short, clear summary of the article's findings; what the study adds to existing research and where and why the results may differ from previous research; strengths and limitations of the study; implications and next steps for research, clinical practice, and/or public policy; one-paragraph conclusion.*

Response: The discussion has been revised to follow this structure

*Please begin this section with “our results show..” or similar, especially at the start of the sentence “The GoActive intervention is not cost-effective”*

Response: This has been revised to:

P19, line 474: “The results of the GoActive trial show that adolescents who participated…”

P19, line 481-482: “The findings also indicate that the GoActive intervention is not cost-effective,…”

*You mention focus group interviews here but these are not fully articulated in earlier sections. This may not be the focus of the current manuscript but please do mention this briefly in the methods section.*

Response: The section of the methods describing the process evaluation measures assessed has been revised to describe more clearly what was assessed.

*Reflections and implications sections should be reduced or removed, since much of this is quite speculative.*

Response: The discussion has been revised and the focus of these sections is now on implications for research and practice. The reviewers commented positively on the discussion (for example, Reviewer 1 states “The discussion provides an honest account of the findings. The authors provide a useful summary of the challenges faced by researchers as they scale-up successful pilot studies.”) and we have therefore not revised this section substantially.

Please complete the CONSORT checklist and ensure that all components of CONSORT are present in the manuscript, including how randomization was performed, allocation concealment, blinding of intervention, definition of lost to follow-up, power statement.

**Reviewer #1:**

*General comments*

*The manuscript describes findings from the GoActive physical activity intervention for adolescents. Physical activity declines dramatically during adolescence and innovative strategies are needed to engage adolescents in health enhancing physical activity. The GoActive intervention was carefully designed and based on a successful pilot study. Study strengths include the rigorous study design, high quality outcomes, large sample size and high consent rates, inclusion of longer-term follow-up assessments and assessment of cost effectiveness. Despite these strengths, the intervention had no effect on reducing the decline in physical activity typically observed in adolescent populations. The authors provide an honest account of their findings and some useful suggestions for moving the field forwards. I provide a list of minor suggestions for improving the manuscript.*

Response: Thank you for your positive feedback and helpful suggestions to improve the manuscript.

*Specific comments*

*INTRODUCTION*

*In general, the authors have provided a strong rationale for their study. Physical activity declines dramatically during adolescence and schools represent an ideal opportunity for intervention.*

*METHODS*

*Study design and participants*

*The study design and methods are rigorous and appropriate for the study aims.*

*Excellent consent rate for students.*

*From the 103 schools that were invited, 16 were successfully recruited. Did the research team track reasons for schools' non-participation? Could this information be included in the CONSORT flow diagram (this is mentioned on page 25 of the protocol)?*

Response: the large majority of schools did not respond to our invitation to participate and we were unable to get hold of a suitable school contact in our follow-up phone calls. For the small number of schools that we were able to connect with, reasons provided included ‘lack of time’, and ‘already doing enough about physical activity’. Two additional schools agreed, but dropped out prior to baseline assessments due to lack of time; these were replaced with two back-up schools we had recruited. We have included this information on p14 (line 366-369). Due to the COVID-19 pandemic, we are currently unable to access the MRC Epidemiology Unit Private Network that holds the database containing this information. This is being resolved, but the timeline is unclear. We have therefore not been able to include the exact numbers in the CONSORT flow diagram, but will strive to include the information should the editor wish to see it included.

*Were schools match paired according to the four variables before randomisation? See Murray text below:*

*Murray, D. M. (1998). Design and analysis of group-randomized trials. New York, NY: Oxford University Press.*

Response: 4 schools were recruited within each combination of pupil premium group and county. Within each of these sets of 4 schools, 2 were randomised to the control group and 2 to the intervention group. No specific pair matching of schools was applied in the randomisation.

*Procedures*

*Self-determination theory (SDT) has emerged as a useful theory for explaining and promoting physical activity in adolescent populations. However, the use of external rewards for participating in physical activity is not consistent with the tenets of SDT for promoting self-determined motivation. Such an approach may have a short-term positive effect, but is unlikely to promote sustained behaviour change. Please comment.*

Response: We state in the introduction that the GoActive intervention was co-designed and not solely based on one theory. We have revised this sentence in the introduction to clarify that not all components of GoActive align with specific theories:

“Although GoActive is broadly aligned with Self-Determination Theory [22], our priority was to co-design the intervention with students and teachers. Therefore, we used theory flexibility to enable the incorporation of components strongly suggested in the development work, irrespective of whether they aligned with theory, such as rewards [13].” (p6, line 130-134)

We have also revised the abstract to reflect the fact that GoActive was aligned with SDT, not fully based on SDT P2, line 50).

*Statistical analyses*

*Did the research team conduct two types of analyses (i.e., complete case and intention to treat)? The following sentence appears contradictory- "We conducted a complete-case analysis based on the Intention to Treat principle, in which participants and schools were included in the group to which they randomised, although participants with a missing value of an outcome at follow-up were excluded from the analysis of that particular variable.". From my understanding of ITT, participants should be included in the analysis regardless of whether or not they completed follow-up assessments. Please elaborate.*

Response: The reviewer is correct that an analysis in which participants with missing outcome data are excluded is not a strict ITT analysis, but as White et al (2012) [1] point out, there will almost always be some loss to follow-up in a clinical trial and therefore “such an unattainable standard is unhelpful”. Because of the missing outcome data, we deliberately referred to the ITT principle, i.e. the inclusion of participants and schools in the group to which they were randomised, rather than an ITT analysis. However, to avoid confusion, we have removed any reference to ITT from the paper; this is in line with CONSORT 2010 [2], which has “dropped the specific request for ITT analysis in favour of a clear description of exactly who was included in which analysis”. (changes made on p13, line 341, and p18, line 443)

*Did the authors test for clustering of effects at the class level? Although participants were randomised at the school level, it appears as though aspects of the intervention were delivered during class-time.*

Response: Accounting for clustering at class level was not specified in the Trial Analysis Plan and we therefore did not include this. This was decided a priori based on the observation in feasibility and pilot work that in UK secondary schools tutor groups (the unit of intervention delivery) can be horizontal (including only students of a single year group) and vertical (including students across the year group). In the latter situation, schools were free to set up an alternative delivery mode, which we could not account for in analyses.

However, in responding to the reviewer’s comment, we have calculated the ICC for the primary outcome for the school-level clustering, which is 0.053. This suggests that the effect of clustering within schools is very small; having taken into account clustering within schools, the effect of clustering within classes is likely to be even smaller. We have therefore not revised the analyses presented in the paper.

*RESULTS*

*If possible, please report the adjusted difference between groups for the psychosocial and anthropometric outcomes in a table (not in a supplementary table).*

Response: We decided to present the intervention effect for the secondary outcomes in a figure to avoid inclusion of too many tables. For the interested readers, we provide the details in the supplementary information (S5). We would prefer to continue presenting the figure instead of the table, but could change this if the editor believes that to be preferable.

*DISCUSSION*

*The discussion provides an honest account of the findings. The authors provide a useful summary of the challenges faced by researchers as they scale-up successful pilot studies.*

*The lack of alignment between reward provision and SDT should be addressed in the discussion.*

Response: As described in response to this reviewer’s earlier comment, the GoActive intervention was not solely based on SDT, but was co-designed and informed by multiple theories, as well as pre-trial research with pertinent subgroups. We have therefore not included this in the discussion.

**Reviewer #2:**

*This paper reports a cluster trial of an intervention to promote physical activity in young people. I was asked for a statistical report and I interpret that to include all aspects of the design and conduct of the study. The version I received did not have page numbers. My numbering has the abstract starting on page 2.*

Response: Apologies for the lack of page and line numbering. This has been corrected in the resubmitted version and we include reference to the specific location of our revisions in this response.

*Points of detail*

*Page 8 What is BMI SDS? I know what BMI is obviously. Is this what the text refers to as a z-score?*

Response: This has been revised to BMI z-score (p15, Table 1)

*Page 9 If I understand the imputation correctly this was a form of single imputation. Since substantial proportions seem to be missing at 10 months this seems unwise as it will underestimate the variability. Some form of multiple imputation would be much better and also some expansion of the description here especially if I have not understood correctly.*

Response: We apologise for this confusion, we have not imputed outcome data for those participants with missing data at 10-month follow-up. Instead, we only imputed sleep times for the nights where this was missing to optimise data use and avoid inflation of our average daily values. We tested this method by running simulations on excluding night data on subsample of compliant data to test the method, which provided satisfactory results. We have now clarified this in the PA data methods on p9.

*Page 10 Categorising an essentially continuous variable like socio-economic status wastes information (Altman and Royston, 2006; Royston et al., 2006) and leads to models which are often implausible as they predict the effect remaining at within categories and then jumping to a new value at the category boundary. Surely a continuous variable could have been used in the interaction term?*

Response: The FAS is an ordinal variable and a proxy measure of socio-economic status. Ideally, we would have collected data on continuous measure such as family income, however, this was not feasible due to our data collection methods (whereby we only include adolescent surveys and not parental surveys). We therefore used a well-validated proxy indicator validated for use in adolescent populations (FAS) and, by its nature, it is categorical rather than continuous. The FAS has been utilised in different ways for different analyses. Currie et al (2008) note that “most frequently FAS has been recoded to create low, middle and high affluence groups […], in order to examine the effect of relative or approximate SES position that more easily corresponds with classical SES groupings” [3]. We therefore prefer to retain the categorisation. We have added a reference to the Currie et al (2008) on p11, line 288 as further justification for this decision.

*Page 10 I did not see the secondary outcomes described in Table 1. In fact they only seem to appear in Figure 3.*

Response: All descriptive and analytical data is presented in tables in the Supplementary file (specifically S5-9 Tables). We have decided to retain the information there, but would be happy to move some to the main manuscript if the editor prefers some of this information there.

*Page 12 I would have thought the outcome here was ordered so an ordinal regression would have been more appropriate than a multinomial.*

Response: Whilst checking the data in response to reviewers’ comments, we discovered an error in our data processing for the self-reported physical activity outcome, to which this comment refers. We apologise for this error, which has been rectified. Self-reported physical activity is now analysed as a continuous outcome, not categorical. The revised processing, descriptive data and results have been described in the revised manuscript. This revision has not affected the results or the conclusions drawn. We have additionally checked all other outcome variables and confirm that no further errors were identified.

*Page 16 The baseline N is larger than the 10 month N so the means are obviously based on different samples. I wonder whether this is helpful as the full baseline sample is not contributing to the change score. We do have the change score with its confidence interval of course so this may not matter too much.*

Response: The reviewer makes a good point, and we agree that it is not clear cut which sample to use when presenting the baseline mean. Only including participants who have a value of the outcome at 10 month follow-up in the calculation of the baseline mean would give N=832 (control) and N=953 (intervention), i.e. lower than the N values at 10 month follow-up, because there are some participants with outcome data at 10 months but missing values at baseline. However, these participants are included in the ANCOVA model to estimate the intervention effect using the missing indicator method, as described in lines 328-329 (p13). Unfortunately therefore we have a choice between presenting the baseline means with N values that are either lower or higher than those at 10 month follow-up, but not the same. On balance we would prefer to retain the current approach, so that these statistics are descriptive summaries of all the observed outcome data at baseline and 10 month follow-up. As well as using the missing indicator method, the estimated intervention effect is also adjusted for baseline and the randomisation stratifiers (p12, lines 322-327), so could not be calculated directly from the means at baseline and 10 month follow-up in each group, even if we removed participants with missing values at 10 month follow-up from the calculation of the mean at baseline.

Using just participants with a value of the outcome at 10 months, the mean MVPA at baseline is 35.5 (SD 18.3) minutes/day in the control group and 35.8 (SD 17.7) minutes/day in the intervention group. These are very similar to those reported in Table 2. We hope this provides some additional reassurance to the reviewer on this issue.

*Page 17, Figure 4 Medium/low v high is presumably socio-economic position but that could be made explicit.*

Response: We have revised this figure to include the effect modifier of interest.

*Page 18 I did not understand the sentence starting Observation contradicted*

Response: This referred to the observations conducted as part of the process evaluation. This has been changed to: “Session observations…” (p18, line 456)

*Page 19 I think one of the references to per protocol should be to intention to treat.*

Response: we have re-read the paragraph the reviewer refers to, but believe that the use of ‘per-protocol’ is correct. No changes have been made.

*Page 23 Perhaps ‘largest to our knowledge’ would be safer.*

Response: this has been revised as suggested (p23, line 603)

*Page 34, Figure 2 This figure could do with more explanation. Is acceleration measured in units of minutes per day as the caption suggests? It is usually length per time squared. We could also use a description of the exact time periods here as well as in the text.*

Response: We have amended Figure 2 to clarify that average acceleration is in milli-g, and that in-school was defined as 9am-3pm and weekdays after-school from 3pm. We have also added this information to the supplementary table describing the outcome measures.

*Page 35, Figure 3 Why do we see the post intervention results here for the secondary outcomes but not, as far as I can see, for the primary?*

Response: We hypothesised that an effect of the intervention would be obtained through changes in the psycho-social variables for which we present the post-intervention effects. Therefore, although the 10-month follow-up is the primary outcome assessment, providing information on the post-intervention effects allows the reader to assess whether meaningful change was obtained at an earlier time point in these hypothesised mediators. All other post-intervention results are presented in the Supplementary Information.

*Points of more substance*

*Detail of reporting - The authors make copious reference to their published protocol. I feel that we need more of the detail from that in this paper too. At the moment it is hard in places with awkward cross-referencing. A particular issue is the secondary outcomes which are really obscure. I think we need to know what scale was used to measure each and at least some indication of the ranges of scores to interpret the x-axis in Figure 3. I know some of this detail is in the supplement but surely the outcomes are important enough for the main paper.*

Response: We have revised the methods following comments from multiple reviewers, and clarified the references to the main trial protocol paper and the process evaluation protocol paper. In response to the specific point on the secondary outcomes: we have included the possible range of scores for these in the methods section to aid interpretation of the effects reported in Figure 3.

*The discussion makes reference in several places to the outcome of the process evaluation but this is still in preparation if I read the statement correctly. It does seem a shame to have the results of a mixed-methods study split like this although I know it is often done.*

Response: As highlighted in the discussion, an initial process evaluation paper based on dose received satisfaction of Year 9 participants has already been published, but a second paper is in preparation. Although we agree with the reviewer that we would ideally present the results together, we believe it would be difficult to do justice to the breadth and depth of data collected as part of the process evaluation if they were only to be presented in this main outcomes paper. Other reviewers have also commented on inclusion of the process evaluation data, but there is variation in what is suggested to be included or not. We have therefore retained a similar level of information as provided in the original submission, but have incorporated revisions to provide more clarity.

*Interpretation - Since the study did show at least some effects in the moderator analyses the treatment must have had some effect even if not the desired one. Moderator effects being interaction are less precisely estimated as well which strengthens the point. Does this affect our view of what the study actually found?*

Response: As defined in our analyses plan we tested for effect modification, and subsequently drew tentative conclusions on differential effects based on the subgroup analyses. We include a discussion of the interpretation and implications of the differential effects observed (pages 21-22)

**Reviewer #3:**

*This paper reports on the evaluation of impact of the GoActive program on adolescent's total daily MVPA across 10 months, noting that the intervention was the most intensive during the initial 12-week period. The study was conducted in the UK, with a somewhat diverse population but does not necessarily include a large sample of racially/ethnically diverse children, low-income children, nor a high percentage of overweight/obese children, who might generally benefit more from school-based interventions. This was a randomized and blinded study in a large population of children and the authors are to be commended for that. However, throughout the manuscript I found that the writing was not necessarily compelling nor specific. I also think that based on their findings that there is the abrupt and somewhat overstated conclusion that we should give up on school-based PA interventions and that they are not cost-effective. That was not the primary goal of their study and it is indeed unfortunate that the results were not more positive. However, there are questions with regard to some of the analyses, the dose delivered, etc. which might resulted in the inability to find a positive effect on PA. We need to remember that we are working to prevent a natural decline in activity over the childhood years and it indeed may become ever more complex during the adolescent years when academics become more competitive and there more of a loss of light activity and a general increase in sedentary time. Fueling self-efficacy around PA might be indeed very important and maintaining the access to PA. In this study, given the focus on total daily activity it would have also been nice to hear more about the out-of-school time activities of these children - the % engaged in sport, etc. Even data on those who walked to school, vs. other means.*

Response: Many thanks for your comments and reflections. We respond to your specific comments below.

*Title: "effectiveness" and "cost-effectiveness" seems a bit redundant/wordy in the title - consider rewording.*

Response: We believe this is a common way of presenting the results of a trial that focusses on both effectiveness and cost-effectiveness, and we consider it a key strength of the project that we want to communicate in the title. In addition, the editor and other reviewers have not commented on the title. As such, we have retained the title as it currently stands.

*Abstract:*

*Introduction: "and social disadvantage" seems a bit misplaced - social disadvantage from chronic disease risk or just from alone not being active?*

Response: “Social disadvantage” refers to the risks associated with lower physical activity to academic achievement and social development. To clarify, we have revised the sentence to: “Adolescent physical activity levels are low, which comes with social disadvantage and rising disease risk at increasingly early ages.” (p2, lines 33-34).

*Methods:*

*Should "Year 9" be capitalized? - if so, your second mention is not.*

Response: We have consulted the UK Department for Education’s Writing Style Guide, which states to capitalise “a particular year level of students, eg Year 7, Year 11 students” (p8). Based on this, we use “Year 9” throughout, and have checked the whole manuscript to ensure consistency.

*It is not clear what N=165(62) and n=193(43) refers to*

Response: This refers to the mean number of recruited participants per school in control and intervention schools, respectively. We have revised this sentence to clarify what the numbers refer to. P2, lines 47-48: “(control: 8 schools, 1319 participants, mean (SD) participants per school: n=165 (62); intervention: 8 schools, 1543 participants, n=193 (43))”

*Introduction*

*Overall, the introduction is somewhat disjointed and does not read smoothly. It seems a bit thrown together and does not exactly prepare the reader for where the authors are at in terms of presenting the current work.*

Response: Thank you for this feedback and the more specific feedback below. Following all reviewers’ feedback, we have substantially revised the introduction, which we hope deals with the reviewer’s concerns.

*First paragraph*

*First sentence - do these data represent in a decline in adolescent physical activity from when to present? Or are you simply implying that globally adolescent PA is low - I would clarify be very precise with this compelling data.*

Response: Based on the reviewers’ comments, we have substantially revised the introduction. This particular sentence now reads: “In adolescence, physical activity levels are low. Recent data shows that less than 20% of adolescents meet the WHO physical activity guidelines of 60 minutes of moderate-to-vigorous physical activity (MVPA) every day, with little change over time [2].” (p2, line 98-101)

*Please clarify what "life chances" are*

Response: This has been changed to “academic achievement” to align with the reference provided (p2, line 103).

*Note that the drop in 5min/day from adolescence to adulthood may not seem that compelling when it is known that children are already dropping on average two minutes per day throughout each progressing year of elementary school….such that levels are already so low. And please just clarify - is this comparing a 17 year old to an 18 year old? Again, some specifics might be more compelling for the reader.*

Response: In our revision of the introduction, this sentence has been removed as it wasn’t deemed necessary to build up the argument for the GoActive trial.

*Paragraph 2*

*Please cite the reference demonstrating that most PA declines throughout adolescence occur outside of school. This may vary substantially on the socio-demographics.*

Response: We have now included a reference to Brooke et al, JSAMS 2014 [4] to support this statement. Interestingly, little research is available on which subgroups of adolescents demonstrate more or less decline at particular times of the week, and so we cannot comment on this suggestion.

*The thought-stream of this paragraph is not fluid. It is difficult to follow the points that are attempting to be made through discussing all of the reviews - are you attempting to highlight the lack of interventions in adolescence? Or are you high-lighting that school-based interventions are ineffective in increasing total daily activity…or other?*

Response: This paragraph has been revised (p5).

*Paragraph 3*

*Needs a transition to this paragraph shifting the reader to the focus of the current intervention (that is not clearly stated). There are too many ideas/concepts in this paragraph - theory development, prior pilot work, the aim of this study, and why it overcame limitations. Some of that information could even be put in the discussion. Finally, the concept of cost-effectiveness feels a bit thrown in and does not have any background or rationale (although understood) in the text.*

Response: This paragraph has been revised (p5/6).

*Also, please spell out moderate-to-vigorous PA the first time it is used (MVPA) and also be precise that the main outcome is total daily MVPA and not school-time*

Response: We have now included the acronym and specified that the main outcome is whole-day MVPA (p5, line 101 and p6, line 138).

*Methods*

*Baseline assessments Sept - Jan?*

Response: The baseline data collection was indeed conducted between September 2016 and January 2017. We are unsure what the reviewer is referring to, but can clarify that we had a large research team and a high number of monitors to enable a high through-put data collection. No revision to the text has been made.

*Accelerometry*

*Why did the criteria for accelerometry data inclusion not include both weekend and weekdays?*

*Inclusion criteria for 6 hours of wear time over at least two days seems very sparse. Would like to see references to support methodology chosen. The protocol, as written, is difficult to follow.*

Response: We have stated in the protocol paper “Monitor output will be reviewed prior to analysis to confirm that these decisions are appropriate for the population and monitor applied.” (Brown et al, 2017, p5) [5]. Following data review, we made the decision to include participants with 2 days of valid data without a requirement for inclusion of a weekday to maximise data inclusion. Of 2638 baseline participants providing two days of valid data, 88.8% provided two weekend days, and 96.7% on at least two weekdays. Re-analysis including those participants with at least one weekday and one weekend day did not show an impact on the findings or conclusions drawn. Our approach aligns with the methods applied in the GirlsActive trial, which also utilised wrist-worn accelerometry and included participants with at least 2 days of data without requiring a weekend day [6]. More recent research also suggests that inclusion of weekend data does not improve the reliability of wrist-worn accelerometry in young people [7]. We have now included both these references in the manuscript. We have additionally re-ordered this section to better clarify the process followed.

*Reported outcomes*

*How can individual socio-economic position be self-reported by this group of children?*

Response: Ideally, we would have collected parent-reported data on socio-economic position, however, this was not feasible due to our recruitment and data collection methods (whereby we obtained parental opt-out consent, and only include adolescent surveys and not parental surveys). We therefore used a well-validated proxy indicator validated for use in adolescent populations (FAS) [3].

*Anthropometry - understand the subject burden of assessing this measure, however I think it is an unfair statement to say that it was not important to assess at 10 months. There would not be an expectation of weight change, however, the differential impact of programming between weight status categories would be an important outcome to assess and understand.*

Response: We apologise if this was not clear. We did not assess anthropometry at post-intervention for the reason stated, but anthropometry was re-assessed at 10-month follow-up and included as a secondary outcome. We have revised this sentence to clarify. It now reads: “As a change to the published protocol, anthropometry was not assessed immediately post-intervention to reduce measurement burden on schools and participants and because no meaningful impact on anthropometry was expected short-term.” (p10, lines 256-259)

*Economic analysis - more explanation of the measures and costs incurred during the program implementation are needed here.*

Response: We have clarified the cost inputs used in the analysis (methods, economic evaluation) (p11-12)

*Process evaluation*

*Please briefly describe the levels of dose/measures survey/inquired about here and what your main process measures are that are utilized in the present study/analysis.*

Response: We have revised this section to describe the process evaluation measures presented in the results more clearly. We also include the relevant questionnaire items in the supplementary information.

*Analyses*

*More information is needed on the physical activity survey and how the variables of relative to never active were determined.*

Response: We have included more detail on this in the manuscript and the supplementary information. Please note that whilst checking the data in response to the reviewers’ comments, we discovered an error in our data processing for the self-reported physical activity outcome. We apologise for this error, which has been rectified. The revised processing, descriptive data and results have been described in the revised manuscript. This revision has not affected the results or the conclusions drawn. We have additionally checked all other outcome variables and confirm that no further errors were identified.

*Please define what "active during tutor times at least twice during the last two weeks" means. How active? For how long? Is this only during the school day or could it be other? Etc.*

Response: We used the process evaluation data collected mid-intervention to define our per-protocol population. Here, the students self-reported the number of times they were active during their tutor time (which happens during school) in the past two weeks. In line with the spirit of the GoActive intervention, this did not specify an intensity or duration. We used this as an indicator of fidelity to the delivery of GoActive, which was intended to be predominantly delivered during tutor time. We have now clarified that this refers to “self-reported intervention engagement mid-intervention” (p13).

*Also be more specific about what activity points are and how often children were instructed to log - is this a daily, weekly, or monthly calendar, etc?*

Response: Activity points were gained every time students tried an activity with no expectation of intensity or time. Activity points were logged on the GoActive website, and the accumulation of points results in rewards. Students were not told how often to log these, but were encouraged to log on regularly so they wouldn’t forget. We have added further detail on the intervention, including activity points, to the relevant section of the methods (p 7-8).

*Results*

*In general, the results described are very vague and do not include much data. It is hard to understand the findings/results in the current written form.*

Response: We have revised the results section by including headings, changing the structure, and have included more detail on the results for the secondary outcomes. However, we are reluctant to include selected quantitative results when all results are fully presented in the tables and figures. We believe that this way the text and tables/figures are complementary, but we would be happy to revise this approach if the editor requests.

*Please describe the population in general terms in written results - age, % female, % overweight, % obese, % non-white.*

Response: We have now added this (p15, lines 382-384): “Overall, participants were 13.2 (SD: 0.2) years, 52.1% were male, and 84.7% were self-reported as White.”

*Primary outcome - results "appearing to favor" seems like very vague language for the results section and should be presented more precisely and with or without significance.*

Response: We have slightly rephrased this sentence (“generally favoured”). However, as stated in an earlier response, we are reluctant to include selected quantitative results when all results are fully presented in the tables and figures.

*Tests for interaction - similarly the language used is very vague…"suggest"…and results are inconclusive.*

Response: Following recent recommendations (e.g. [8]), we have not based our conclusions on the significance (p-value) of the effect estimates, but instead consider the effect estimate and confidence interval. As also noted by reviewer 2, interaction and subgroup effects are generally estimated with less power, which we have also taken into account. We have rephrased this paragraph to clarify, but consider it important to highlight these important differences in a considerate way (p18).

*There is general concern that the per-protocol analyses only included 382 children and that the inclusion criteria for following the protocol did not seem to necessitate a very large dose of activity delivered.*

Response: We agree with the reviewer that this is a concern, and an indication that the intervention was not delivered with high fidelity. Prior to analyses we reviewed the process evaluation data to determine the per-protocol group, aiming to achieve a balance between sample size and fidelity. As schools were flexible in how and how frequently GoActive was delivered, this is therefore not a measure of what ‘should have been delivered’, but an identification of the group that received the highest dose delivered. To clarify, we have added the following to the methods: (p13, line 350-353): “This definition was based on a review of quantitative process evaluation data prior to the main analyses, and reflects the group with highest intervention engagement as opposed to delivery of the protocol with fidelity.” We have also commented on the low sample size for the per-protocol analysis and low fidelity in the discussion.

*Process evaluation*

*A better understanding of what it means for students to have a peer-leader vs. a tutor, etc. is needed.*

Response: We apologise that the terminology used was not clear. A tutor is a normal part of the English secondary school system and is a member of teaching staff responsible for a group of students, usually in the same year group. In GoActive, tutors supported the intervention, but GoActive was developed to be delivered by mentors (students from older year groups) and peer leaders (fellow Year 9 students). We have now clarified this in the text (p7-8). We have additionally published on adolescents’ experiences in our first process evaluation paper, entitled “Adolescents' perspectives on a school-based physical activity intervention: A mixed method study.” [9]. This highlighted the lack of implementation of in-class peer leaders. Adolescents also described that mentors and teachers seemed confused with their roles, likely affecting the implementation. We have now mentioned this in the discussion as well.

*Figures.*

*Legends need to describe what is being shown in more detail. They currently do not stand alone. What is acceleration?*

Response: We have revised the figures to provide more detail on what they represent. Average acceleration is the mean acceleration measured by waveform accelerometry in milli-g.

*Is self-efficacy - general or PA self-efficacy?*

Response: We assessed PA self-efficacy, this has now been clarified in the text and supplementary file.

*Discussion*

*Why does the first paragraph end with the notion that the intervention could have negatively impacted well-being?*

Response: One of the aims of GoActive was to assess whether there were any negative effects on mental well-being as a result of the approach employed. We have now included this as a limitation of previous research in the introduction (p6).

*If baseline was Sept-Jan then 10month follow-up occurred between the months of? July-November? That doesn't really make sense given the academic year. The discussion also mentions across "two academic years".*

Response: We aimed to assess the main outcome on average 10 months post-intervention, not post-baseline. 10-month follow-up was therefore conducted between April and June 2018, which is when students were in Year 10. We have clarified this in the methods (p8, line 199).

*There was also no mention of accounting for the time of year of measure at baseline vs. followup. (or weather for that matter)*

Response: This is correct. Schools were all randomised at a similar time of year, and in any case we would not expect any systematic difference in the weather between the randomised groups, so we did not consider it necessary to account for either of these factors.

*What was the children's average school-time physical activity? How much did this decline over the time-frame? Vs. total daily? And did you indeed see more of a decrease out of school time? I think that the discussion is too focused on weekend days and out of school time, when the intervention was school-based.*

Response: We present all the descriptive data for the accelerometer-based secondary outcomes at all three time points in the supplementary file (S5 Table), and the results of the analyses are presented in Figure 2, S6 Table, and S7 Table. To avoid having too many tables in the main manuscript, we prefer to retain this information here. As Figure 2 shows, the intervention effect was differentially distributed across the week, with a more favourable effect estimate at weekends as opposed to during or after school. We have previously argued that studying the distribution of the intervention effects across the week is important to gain an understanding of how the intervention may work [10], and we believe it is appropriate to consider these in our discussion. A paragraph discussing differential effects across the week is included on p21.

*Limitations*

*Failure to acknowledge some of the variables that could have been assessed such as longer wear-time inclusion criteria for accelerometry, weather, time of year of the different visits, child measure of SES, specified dose of delivery for children to achieve in school, and somewhat of a lack of lower-SES and racial/ethnic diversity.*

Response: We acknowledge that this trial comes with its limitations. We point out that we conducted post-hoc sensitivity analyses in which the primary outcome was calculated (1) excluding time between midnight and 6am (2) using a stricter inclusion criteria for wear time of 12 hours of wear per quadrant. The results of these are presented in the results section and S10 Table, and did not differ from the main results. As noted earlier, we do not consider accounting for weather and time of year of visits appropriate in the analyses of a randomised controlled trial.

It is important to recognise that decisions about study design come with trade-offs in what can be collected. Our decision to obtain parental opt-out consent has resulted in a very high recruitment rate (84.1%) of a population that is broadly representative of the population it was drawn from, but this prevented us from collecting parental-reported data (such as income) or (semi-)identifiable data (such as postcode). We included a well-validated and previously used measure of adolescent-reported socio-economic position, but acknowledge that this may differ from parent-reported socio-economic position. We have included this, the relative lack of socio-economic and racial diversity, and failure to deliver the intervention with fidelity as additional limitations (23, line 592-593).

*Final there appeared to be a disregard in understanding the dose received and uptake by important sub-groups such as girls and those who are overweight/obese. Discussion of the prevention of decline in minutes of MVPA in girls vs. boys might be very illustrative.*

Response: We are sorry that the reviewer considers that we disregard understanding of dose and uptake. Alongside the GoActive effect evaluation, we conducted an extensive mixed-methods process evaluation to understand the process of intervention delivery and to provide context to the quantitative effect findings (see [11] for published protocol). In a previously published process evaluation paper, we report that girls did not enjoy the programme as much as boys, and that there was observed lack of engagement from girls [9]. A more detailed process evaluation paper considering dose is currently in preparation. The revised discussion more detail on gender-specific findings to address the differences between the process evaluation findings and the effects observed (p21-22). As suggested in the response to another reviewer, although we would ideally present the results of the effect and process evaluation together, we believe it would be difficult to do justice to the breadth and depth of data collected as part of the process evaluation if they were only to be presented in this main outcomes paper. Other reviewers have also commented on inclusion of the process evaluation data, but there is variation in what is suggested to be included or not. We have therefore retained a similar level of information as provided in the original submission.

*Given the apparent lack of dose delivered and received by the adolescents in this study, this reviewer believes that it may be somewhat of a premature assumption and editorial by the authors to state that we should divert our resources elsewhere beyond the promotion of physical activity in schools. Multiple modalities are warranted and indeed continue to be very difficult to assess in a natural way, but in a robust clinical trial design with adequate statistical rigor.*

Response: We have revised the conclusion following multiple reviewers’ comments (p25-26).

**Reviewer #4:**

*This paper reports of the effectiveness and cost effectiveness of a large cluster RCT in the UK. Like a number of other well-designed UK school-based trials the intervention aims to use peer leaders to influence younger pupils to become or stay active with the hope of reducing the age-related decline seen. A number of papers related to this study have already been published, including the protocol paper. Despite the preparation for this definitive RCT being linear with clearly related outputs (i.e. development work, feasibility study, protocol paper) the presentation in this paper is not wholly clear. The paper could do with a rework in how the work leading up this this RCT is presented, the clarity with which the actual intervention is presented and the rationale for including process evaluation peppered around the paper. Well done on the whole study and getting it down on paper here, they are a lot of work!*

Response: Many thanks for your encouraging comments and your thoughtful feedback.

*Critical comments:*

*Process evaluation section is a bit lost in there and doesn't add anything especially if more triangulation work or reporting is planned in a separate paper. I would suggest removing and just sticking to primary outcomes and the costs.*

Response: Other reviewers have also commented on inclusion of the process evaluation data, but there is variation in what is suggested to be included or not. We agree that it is difficult to do justice to the breadth and depth of data collected as part of the process evaluation in this main outcomes paper. On the other hand, some of the process evaluation findings are key to understanding the main outcomes results, and are embedded in our interpretation, and conclusions. Inclusion of some of the process evaluation therefore strengthens this work and to not use the findings here to help explain the results would be a wasted opportunity. We have therefore retained a similar level of information as provided in the original submission, but have revised how the process evaluation data is presented.

*The authors state the programme was not cost-effective. Firstly, the programme was not effective at changing the primary outcome so cost effective analysis is inappropriate. Should this be a cost consequence analysis? This would describe what the cost consequences of delivering the intervention were?*

Response: We respectfully disagree with the reviewer’s comment. An intervention can be less effective than a comparator (or as effective) yet be cost-effective if it is substantially less costly: foregoing a small quantity of effectiveness (e.g. QALYs) could release a large quantity of resources which can be put to better use elsewhere, increasing overall net health gain for the resources. We believe the reviewer suggests a cost minimisation analysis (CMA), which considers cost only, as a cost-consequences analysis is simply reporting disaggregated costs and outcomes in each arm, without calculation of ratios. CMAs are generally advised against as they interpret absence of evidence as evidence of absence: ignoring outcomes assumes they are identical, whereas we do not have any proof that the ‘treatment effect’ is exactly zero. Ignoring outcomes therefore underestimates uncertainty in cost effectiveness (see for example Briggs & O’Brien 2001 “The death of cost-minimisation analysis?” [12]).

*Economic analyses of trials like this are in their infancy in the UK. But now we have a number of large NIHR trials including ones from this PHRP call that have used economic analysis (also WAVES) that is building up since methods, tools and data in this area. Have you considered a separate paper on this? One that includes methods, reflections etc.*

Response: We thank the reviewer for this suggestion. We had not considered this, but an overview of the evidence and methods development could well be a useful addition to the literature.

*Major comments:*

*1. The authors stated the feasibility was promising. What was the difference between the feasibility and this RCT? The authors state that the engagement with online and was low and "contrasts high engagement in the pilot trial" but was there anything related to the delivery that was different? I think this is a key piece of the puzzle of how promising feasibility/pilot does not always translate into effective main trials. Consider (even in a separate short report) a table of differences between feasibility and the main trial in terms of, for example, delivery (type, amount, staff, training), support provided by the research team, the "buy in" from the delivery staff (vs. delivery from vested researchers) etc etc.*

Response: We believe the reviewer is referring to the findings of the pilot study, which were indeed promising. As highlighted in a recent publication by Beets et al [13], ‘voltage drop’ between pilot trials and larger effectiveness trials is very common in health promotion research. They identified delivery agent, implementation support, duration, and measurement bias as key contributors to the reduced effect observed in full trials as opposed to in pilots. Out of these, the key change to implement in the GoActive main trial was delivery of the intervention through an outside agency, supported by the main research team. We have now commented on this change in the discussion (p20).

*2. Paper goes very quickly from the lit into the background on the trial (top of page 13). A clear opening sentence introducing the programme would be useful there. The section starting with the following blends a little too much with the background/intro to the intervention. "Previous research suggests that activity promotion strategies should be based on behaviour change theory, existing evidence and pre-trial qualitative work with the target group (13, 17). Although the intervention was broadly aligned with self-determination theory (18), our priority was to co-design the intervention with students and teachers. Therefore we used theory flexibility to enable the incorporation of components strongly suggested in the development work, such as rewards." Suggest the whole section be split. Deal feasibility data separately as it is all mixed into one.*

Response: Based on all reviewers’ comments, we have substantially revised the introduction.

*3. In relation to the delivery staff from Active Essex and Everyone Health. How sure are you that the delivery was as robust and fidelity compared to the feasibility? In outsourcing our intervention delivery to suit the funding call/stream (which is a positive to promote sustainability!) are we underserving the original intervention? What did the delivery staff say? What do Active Essex and EH think of the programme, will they keep it on their books?*

Response: We intended to study effectiveness, not efficacy, which comes with challenges of real-life delivery. Satisfaction of the intervention, from the perspective of the local council facilitators (Active Essex and Everyone Health), will be presented in the upcoming process evaluation manuscript. As part of the full process evaluation, we conducted interviews with the local health trainers (amongst others), which suggests that they did not deliver their element as suggested. In the training they were all enthusiastic but then life and their other work got in the way, and many of them left throughout the process. This suggests that outsourcing delivery as conducted as part of GoActive was not realistic given the structure / turnover of staff in those institutions. We prefer to give a more detailed account of these findings in the process evaluation paper and have not made changes to the manuscript.

*4. Sentence on "Prior to April 2018, schools received money for every child whose families received income support and had an annual gross income of £16,190 or less(22)." Seems lost and I would say not needed as you have given the reference in the previous statement.*

Response: We removed the sentence as suggested.

*5. Reference number 13 has been used quite a bit and is the reference for the intervention development work. This is not stated anywhere in the paper. I think you need a clearer section about the development of the intervention, the feasibility trial and the feasibility results. This can go in the intro and will help "set the scene" for this definitive cluster RCT. As it stands I am not sure what background info has come from the literature and what has come from the team's own development work. I feel the intro to the development work is undersold!! State that reference 21 is the protocol.*

Response: We now include further detail on the GoActive development work in the introduction to provide further background to the project.

*6. The methods state that in the recruitment areas "substantial socioeconomic diversity and includes both urban and rural areas" existed. Consider whether this is enough to account for the context that schools exist in. "Each school is a unique system" very true. The context is key. Will this be explored in the process evaluation?*

Response: The full process evaluation will consider the culture of the school in relation to physical activity, but not the wider physical environmental context within which the school sits (e.g. walkability, urban/rural). Due to the low number of schools, we are unable to study whether effects differ by urban/rural location of the school, and to our knowledge there is no evidence that physical activity interventions are more or less effective dependent in urban or rural environments. We have clarified the focus on school micro-environment in the discussion (p24, line 626).

*7. Results table: I have not seen a %missing column within the table, usually in the footnote. If it has to go in I suggest at the third column. Optional.*

Response: We have added this information to the notes below Table 2.

*8. Costs collection were supplemented by study records, Give examples of what these study records were. Who were responsible for keeping these updated?*

Response: These were study records on facilitators’ travel expenses and materials. As per comments from reviewer #3, we have added comments to the methods/economic analysis section.

*9. When describing the primary outcome of PA at 10 months. Be clear that that is the accelerometer measure of PA because the next section talks about self-report.*

Response: This has been clarified (p16, line 407).

*10. SES of the ppt was used in the sub-group analysis. Please add a justification. Would that mean that if an SES effect was seen that interventions would be tailored depending on a ppt SES?*

Response: We investigated differences in effect by individual-level SES as we were interested to see if sub-groups of students benefitted more or less from the GoActive intervention. A differential effect does not necessitate tailoring of future interventions, but provides an indication that groups of individuals engaged more or less with the interventions and that strategies should be developed that are more inclusive. We have now provided a justification for our choice of effect modifiers in the relevant section.

*11. Economic analysis - where do those costs lie with in the current delivery i.e. how much of that cost was bourne by the school (printing, cost of teacher time prepping or delivering), bourne by the research team but presumably would have to be paid for by a commissioner in the future?*

Response: The study perspective was that of the school funder, which in England is ultimately the Local Authority. For the purpose of the study, the research team did incur costs, but the costing was analysed as if the intervention were to be rolled out by a Local Authority, so travel expenses etc., whilst on occasion extracted from study financial returns, were applied as if charged to the Local Authority. We have added a clarification to first paragraph of Methods/Economic analysis (p11-12).

*12. "There was no evidence that the intervention negatively impacted wellbeing" but this is only based on the measures that were included in Table S1. Restate with caveat.*

Response: This is also presented in Figure 3, and reported in the results text. We therefore believe that this statement is sufficiently supported to be retained.

*13. The paper states that "teachers and mentors found the programme too complicated and we perhaps afforded schools too much flexibility." Does this come out in the PE teacher interviews? This is an interesting point. This tallies with the finding in the Girls Active trial process evaluation whereby "The flexibility created by having choice in activities and timing of delivery on occasion created uncertainty. Without milestones or deadlines teachers found other priorities took over and the programme drifted, potentially explaining why schools did not achieve everything they set themselves in their action plans." Gorely, et al. "Process evaluation of the school-based Girls Active programme." BMC public Health 19.1 (2019). It is a fine balance between them all!*

Response: We have rephrased this section to provide more clarity on the challenges identified, and have included the similarities with the Girls Active experience (p20)

*14. The intervention is based on SDT in relation to the adolescent ppts. What about the teachers? Is there an opportunity to explore how this flexibility relates to their need for using their expertise, getting appropriate social support…anything in teacher interviews about this and mapping teacher responses onto a theory.*

Response: The GoActive intervention was set up to minimise the role of teachers in intervention delivery, instead relying on a tiered delivery system of council facilitator, mentorship and peer leaders in which the teachers would play a support role. We consider it beyond the scope of the current paper to include more detail here.

*15. There is a lot on the PE in the discussion but little in the results. I think this is an error because that process evaluation paper will be standalone and will be good. However, I know it is hard because you want to give readers some context on why the outcomes are the way they are. Perhaps reconsider what and how much of the PE is included herein.*

Response: We have included more detail on the process evaluation results. Additionally, following feedback from all reviewers, we have revised the discussion. As part of this, we have reduced the reliance on the process evaluation results not included in the current paper or already published.

*16. I found the results largely hard to follow. The terms "effect modification" and "subgroups" are included the stats section. "Sub-group" and "test for interaction" are in the results. While meditation and interaction analyses are included in the discussion. There needs to be some consistency in how the terms are used and some description up front as to what both are looking to explore in practice.*

Response: We now use the terms ‘effect modification’ and subgroup where relevant. The mediation analyses have been published [14], however the reference to these have been removed in the revision of the discussion.

*17. The authors state "the intervention may have fostered a climate that was not conducive to physical activity within school" (ref 23) Vague, what does this actually mean?*

Response: We have now provided examples of what this means: (p21): “(for example, the sessions appeared to have a lack of social cohesion and connection, and that activity choice was often dominated by boys)”.

*18. The authors state "our results are relevant to many schools across the UK and to many other high income settings" but what are these results? What is that take home message from this that is relevant?*

Response: This sentence comes from the strengths and limitations section, where we highlight that our sample is broadly population representative. We believe it is important to point out that the findings observed here, even though we were not able to achieve a positive effect on the primary outcome, are likely to be similar in other settings as well. No revision has been made.

*19. In the reflections section the authors state "the lack of effectiveness of school based physical activity promotion strategies" but these are all the ones that have been in research which are either research-driven. But the PHRP interventions are not always researcher driven. They are often a programme running and then adding research on. There are likely plenty of examples of good practice out there that are school specific and are held by teachers or principals and are part of a whole school-approach to health. Was there any evidence of the buy in from the wider school, other teachers, school senior leadership team?*

Response: We have clarified that we refer to mostly research-driven initiatives, although point out that high quality evaluations of practice-driven initiatives, such as Girls Active, have also been unable to demonstrate effectiveness. We agree with the reviewer that a whole-school approach (including senior leadership buy-in) to health promotion has promise, and include reference to the recent paper by Daly-Smith et al (2020) [15] in which they provide a model for how to achieve this, but highlight that the effectiveness of this approach is yet to be established. We will explore the importance of senior leadership on implementation and effectiveness in more detail in the full process evaluation.

*20. The Daily Mile papers are mentioned and how simplicity of that programme is key. But it is also the cost. The fact that it is free is attractive in times of austerity.*

Response: We agree that simplicity and cost are important. However, despite this, recent evidence also indicates large variation in implementation of the Daily Mile, potentially affecting its effectiveness [16]. We also point out that the GoActive programme was free for schools to deliver, as the intervention costs were borne by the local council. We have clarified this in the methods (p11).

*21. The authors state that "Our findings suggest that current research driven approaches to school physical activity promotion are unlikely to be effective" is a sweeping statement. I think taken with the other non-effective trials (even just the others funded through the same NIHR themed call) there is an issue.*

Response: We have rephrased this sentence to better reflect the more cautious consideration of the wider evidence in the discussion (p25-26): “Together with other recent evidence this suggests that research driven approaches to school-based adolescent physical activity promotion are not effective, with implementation challenges likely playing an important role in the lack of effect.”

*22. Again the statement in the conclusion "Authorities should be cautious about commissioning and proliferating school-based physical activity promotion strategies and be realistic about expectations of effect." Is not representative of this work.*

Response: We have rephrased this sentence to be more cautious. (p26: “School-leaders and authorities should be realistic about expectations of effect of school-based adolescent physical activity promotion strategies implemented at scale.”)

*23. The whole discussion needs tightening up but the comments above may help with that.*

Response: We have revised the discussion following feedback from multiple reviewers.

*24. Is "procedures" the best heading for the section about the intervention? Again, that section needs to be tidied up for clarity. I feel there could be a better use of headings within the methods section to guide readers.*

Response: We have changed the heading structure of the methods section, and have renamed the ‘procedures’ section to ‘GoActive intervention’.

Minor/Typos

*1. Is this sentence missing a word "Total accrued time from baseline to 10-month follow-up and hence the time horizon for the study is approximately two academic years."*

Response: We have dropped ‘accrued’ to clarify.

*2. Results. Opening line Figure is misspelled. Called "trial profile"*

Response: This has been rephrased to ‘study flow chart’.

*3. Table 2 title is missing a word*

Response: This has been rephrased: “Results for primary outcome of the GoActive trial: average daily moderate-to-vigorous physical activity (MVPA, in minutes/day) at 10-month follow-up.”

*4. S12. What is T4?*

Response: Across the supplementary tables we have rephrased the terms T1, T3, and T4 to baseline, post-intervention, and 10-month follow-up, respectively.

*5. Figure 4. Medium/low and high. State what subgroup this relates to.*

Response: This refers to socio-economic status, and this has now been clarified in Figure 4.

*6. Underweight is included in S7 but no mention of that anywhere else. What were the numbers in each category? Add to baseline characteristics.*

Response: This information has been added to Table 1. In the effect modification / subgroup analyses underweight and normal weight were grouped together, and overweight and obese were grouped together.

**References in response to reviewer comments:**

1.	White IR, Carpenter J, Horton NJ: **Including all individuals is not enough: lessons for intention-to-treat analysis**. *Clinical trials* 2012, **9**(4):396-407.

2.	Schulz KF, Altman DG, Moher D: **CONSORT 2010 statement: Updated guidelines for reporting parallel group randomised trials**. *BMC medicine* 2010, **8**(1):18.

3.	Currie C, Molcho M, Boyce W, Holstein B, Torsheim T, Richter M: **Researching health inequalities in adolescents: the development of the Health Behaviour in School-Aged Children (HBSC) family affluence scale**. *Soc Sci Med* 2008, **66**(6):1429-1436.

4.	Brooke HL, Atkin AJ, Corder K, Ekelund U, van Sluijs EM: **Changes in time-segment specific physical activity between ages 10 and 14 years: A longitudinal observational study**. *Journal of science and medicine in sport* 2016, **19**(1):29-34.

5.	Brown HE, Whittle F, Jong ST, Croxson C, Sharp SJ, Wilkinson P, Wilson EC, van Sluijs EM, Vignoles A, Corder K: **A cluster randomised controlled trial to evaluate the effectiveness and cost-effectiveness of the GoActive intervention to increase physical activity among adolescents aged 13-14 years**. *BMJ open* 2017, **7**(9):e014419.

6.	Harrington DM, Davies MJ, Bodicoat DH, Charles JM, Chudasama YV, Gorely T, Khunti K, Plekhanova T, Rowlands AV, Sherar LB *et al*: **Effectiveness of the 'Girls Active' school-based physical activity programme: A cluster randomised controlled trial**. *The international journal of behavioral nutrition and physical activity* 2018, **15**(1):40.

7.	Ricardo LIC, Wendt A, Galliano LM, de Andrade Muller W, Nino Cruz GI, Wehrmeister F, Brage S, Ekelund U, Crochemore MSI: **Number of days required to estimate physical activity constructs objectively measured in different age groups: Findings from three Brazilian (Pelotas) population-based birth cohorts**. *Plos One* 2020, **15**(1):e0216017.

8.	Wasserstein RL, Schrim AL, Lazar NA: **Moving to a World Beyond “p < 0.05”**. *American Statistician* 2019, **73**(S1):1-19.

9.	Jong S, Croxson C, Guell C, Lawlor E, Foubister C, Brown H, Wells E, Wilkinson P, Vignoles A, van Sluijs E *et al*: **Adolescents' perspectives on a school-based physical activity intervention: a mixed method study**. *Journal of Sport and Health Science* 2019.

10.	van Sluijs EM, Kriemler S: **Reflections on physical activity intervention research in young people - dos, don'ts, and critical thoughts**. *The international journal of behavioral nutrition and physical activity* 2016, **13**:25.

11.	Jong ST, Brown HE, Croxson CHD, Wilkinson P, Corder KL, van Sluijs EMF: **GoActive: a protocol for the mixed methods process evaluation of a school-based physical activity promotion programme for 13-14year old adolescents**. *Trials* 2018, **19**(1):282.

12.	Briggs AH, O'Brien BJ: **The death of cost-minimization analysis?** *Health economics* 2001, **10**(2):179-184.

13.	Beets MW, Weaver RG, Ioannidis JPA, Geraci M, Brazendale K, Decker L, Okely AD, Lubans D, van Sluijs E, Jago R *et al*: **Identification and evaluation of risk of generalizability biases in pilot versus efficacy/effectiveness trials: a systematic review and meta-analysis**. *The international journal of behavioral nutrition and physical activity* 2020, **17**(1):19.

14.	Corder K, Werneck AO, Jong ST, Hoare E, Brown HE, Foubister C, Wilkinson PO, van Sluijs EM: **Pathways to Increasing Adolescent Physical Activity and Wellbeing: A Mediation Analysis of Intervention Components Designed Using a Participatory Approach**. *International journal of environmental research and public health* 2020, **17**(2).

15.	Daly-Smith A, Quarmby T, Archbold VSJ, Corrigan N, Wilson D, Resaland GK, Bartholomew JB, Singh A, Tjomsland HE, Sherar LB *et al*: **Using a multi-stakeholder experience-based design process to co-develop the Creating Active Schools Framework**. *The international journal of behavioral nutrition and physical activity* 2020, **17**(1):13.

16.	Hanckel B, Ruta D, Scott G, Peacock JL, Green J: **The Daily Mile as a public health intervention: a rapid ethnographic assessment of uptake and implementation in South London, UK**. *BMC public health* 2019, **19**(1):1167.
