# Supplementary material for: Effectiveness and cost-effectiveness of the GoActive intervention to increase physical activity among UK adolescents: A cluster randomised controlled trial
Source: PLoS Med. 2020 Jul 23;17(7):e1003210. doi: 10.1371/journal.pmed.1003210 (PMC7377379; doi:10.1371/journal.pmed.1003210)
Supplement: Attachment — Submitted filename: R5 response_Corder.docx [file pmed.1003210.s022.docx]

Response to additional production queries (email dd 19^th^ June 2020) – R5

*1. Please note that your figures do not currently comply with our publication requirements. For further instructions to assist with your Figure revisions, please refer to our Figure Guidelines at: http://journals.plos.org/plosmedicine/s/figures.*

*If you need assistance, please contact us directly at graphics@jjeditorial.com so we can further help with this matter. Please send any relevant source files and tell us what application(s) and what operating system you are using.*

*-Figures 1-4 look like they were originally at a lower resolution and will appear blurry or pixelated if published. Text and images should appear crisp and clear. [text shortened by author]*

Response: The figures uploaded were kindly re-created by the graphics department. Following further conversation with the editorial team we received the following response on Fri 26^th^ June: “I've had a look at the figures that are currently uploaded and they may come out a little blurry in the final paper, this is usually due to the resolution you have made the figures with. I think we can go ahead with these as they are, but I would suggest you print the figures out on A4 and assess if you're happy with they look, if you are please resubmit the paper and we will should be able to accept it for you.” We have followed this advice and are happy with the way the figures print. We have therefore not uploaded new figures.

*2. Please remove the funding information from the Acknowledgments section of your manuscript file. The published version is pulled automatically from the “Financial Disclosure” field in our submission form and must not be duplicated. Please therefore take this opportunity to check that the information in this field is correct and completed in full.*

Response: We have removed the reference to funding from the acknowledgement section.

*3. Please ensure your references are in the style of PLOS Medicine (ICMJE/Vancouver style). The reference style guide can be found here: http://journals.plos.org/plosmedicine/s/submission-guidelines#loc-references. You can also find a downloadable EndNote file in these guidelines to assist you. Please do a manual check as well, as the EndNote file does not catch everything. Please note that the references will be typeset as they appear so please ensure the format is consistent.*

Response: It appeared we had downloaded a wrong version of the PLOS endnote style. This has now been rectified and we believe that the references are now in the appropriate style. We have also checked the reference list.
